# RNA methylation-mediated LINC01559 suppresses colorectal cancer progression by regulating the miR-106b-5p/PTEN axis

**DOI:** 10.7150/ijbs.70630

**Published:** 2022-04-24

**Authors:** Ke Shi, Shuaixi Yang, Chen Chen, Bo Shao, Yaxin Guo, Xiaoke Wu, Luyang Zhao, Xiuxiu Yang, Qiuge Zhang, Weitang Yuan, Zhenqiang Sun

**Affiliations:** 1 Department of Colorectal Surgery, The First Affiliated Hospital of Zhengzhou University, Zhengzhou 450052, Henan, China.; 2 Department of Plastic Surgery, Central South University Third Xiangya Hospital, Changsha 410013, Hunan, China.; 3 Academy of Medical Sciences, Zhengzhou University, Zhengzhou 450052, Henan, China.; 4 School of Life Science, Zhengzhou University, Zhengzhou, 450001, Henan, China.; 5 School of Basic Medical Sciences, Zhengzhou University, Zhengzhou 450002, Henan, China.; 6 Henan Academy of Medical and Pharmaceutical Sciences, Zhengzhou University, Zhengzhou 450052, Henan, China.; 7 Department of Neurology, The First Affiliated Hospital of Zhengzhou University, Zhengzhou 450052, Henan, China.; 8 Department of Geriatric Medicine, The First Affiliated Hospital of Zhengzhou University, Zhengzhou 450052, Henan, China.

**Keywords:** LINC01559, PTEN, METTL3, colorectal cancer

## Abstract

Long noncoding RNAs (lncRNAs) regulate multiple biological effects in cancers. Recently, RNA methylation has been found to modify not only coding RNAs but also some noncoding RNAs. How RNA methylation affects lncRNAs to affect colorectal cancer (CRC) progression remains elusive. The expression of LINC01559 was explored through RNA sequencing, quantitative real-time PCR (qRT-PCR) and *in situ* hybridization (ISH). The preliminary exploration of its function was performed using Western blotting (WB) and immunohistochemistry (IHC). Functional experiments *in vitro* and* in vivo* were conducted to explore the biological functions of LINC01559 in CRC. The LINC01559/miR-106-5p/PTEN axis was verified through fluorescence *in situ* hybridization (FISH), luciferase assays, and rescue experiments. RIP-sequencing, m6A RNA immunoprecipitation (MeRIP) assays and bioinformatic analysis were conducted to determine the upstream mechanism of LINC01559. The results showed that LINC01559 was downregulated in CRC compared with normal controls. Lower expression of LINC01559 in CRC patients predicted a poor prognosis. In addition, PTEN was found to be positively correlated with LINC01559, and miR-106b-5p could be the link between LINC01559 and PTEN. Then, silencing LINC01559 restored the malignant phenotype of CRC cells, while cotransfection of miR-106b-5p inhibitor neutralized this effect. Mechanistically, we found abundant m6A modification sites on LINC01559. Then, we uncovered these sites as potential targets of METTL3 through experiments *in vivo*. The results revealed a negative functional regulation of the LINC01559/miR-106b-5p/PTEN axis in CRC progression and explored a new mechanism of METTL3-mediated m6A modification on LINC01559. These results elucidate a novel potential therapeutic target for CRC treatment.

## Background

Colorectal cancer (CRC) is ranked third in terms of incidence and is a significant cause of morbidity and mortality worldwide 1, 2. Over 1.8 million new CRC cases and 881,000 deaths occurred in 2018, accounting for approximately 1 in 10 cancer cases and deaths 1. Although the overall survival (OS) of CRC patients has improved with the development of medical technology, the outcome of advanced-stage colorectal cancer patients remains poor 3. Therefore, more effective and efficient biomarkers and molecular mechanisms need to be investigated in both the diagnosis and treatment of CRC.

Long noncoding RNAs (lncRNAs) are a newly emerged class of noncoding RNAs containing more than 200 nucleotides that are widely transcribed in the genome 4, 5. Due to the diverse cellular functions of lncRNAs, they play essential roles in almost every cellular process, including proliferation, differentiation and apoptosis 6, 7. Furthermore, mechanistic studies have shown that lncRNAs can affect chromatin structure and RNA interactions, such as their function as a microRNA (miRNA) sponge, in which lncRNAs can interact with miRNAs through their seed sequences to reduce the miRNA regulatory effect on target mRNAs.

In recent years, the use of lncRNA sequencing for the diagnosis of Mendelian or rare genetic disorders has become routine clinical practice 2. LncRNA sequencing can reveal the dynamic process of gene transcription, which varies according to tissue type, cellular conditions, and environmental factors and may affect regulatory events such as splicing and the expression of genes or their isoforms 8. LncRNA sequencing with cluster analysis of differentially expressed lncRNAs could identify the expression patterns of different genes under different biological or experimental conditions and aggregate lncRNAs with the same or similar expression patterns 9. Information from chromosome distribution analysis could further reveal important relationships with gene functions 10. Therefore, the importance of lncRNA sequencing as a clinical diagnostic tool has increased. Analyses of the differential expression of lncRNAs and their positions on chromosomes found that LINC01559 is expressed at low levels in CRC tissues and located in chromosome mapping to 12p12-12p13.1, which is always absent from mutations in human tumors 11. However, the potential biological functions, characteristics and mechanisms of LINC01559 in CRC progression have not been elucidated in pioneering studies.

N6-methyladenosine (m6A) RNA methylation is one of the most ubiquitous internal modifications on eukaryotic messenger RNAs (mRNAs), accounting for approximately 50% of total methylated ribonucleotides and 0.1-0.4% of all adenosines in total cellular RNAs 12. Previous research has shown that over 300 noncoding RNAs in humans and mice were found through m6A-specific immunoprecipitation (MeRIP-Seq) 12. Moreover, m6A modification in noncoding RNAs could play a critical role in virtually all major normal bioprocesses, including stem cell self-renewal and differentiation, tissue development, heat shock or DNA damage response, maternal-to-zygotic transition, primary microRNA processing, and RNA-protein interactions 12. As lncRNAs are transcribed and modified similarly to messenger RNA (though typically with many more m6A sites), m6A RNA methylation may regulate lncRNAs more actively than what previously believed through a mechanism similar to mRNA.

Mechanistically, The dynamic and reversible N6-methyladenosine (m6A) RNA modification preferentially occurs in the consensus motif “RRm6ACH” (R =G or A; H =A, C or U) and is mediated by m6A WERs (“writers”, “erasers” and “readers”), including the m6A “writer” methyltransferase METTL3 13, 14. A growing number of studies have confirmed the functions of m6A in a variety of malignancies. Nevertheless, the definite role of m6A in CRC remains unclear, and the dysregulation of METTL3-mediated m6A modification in the progression of CRC needs to be further explored.

In this study, the RNA sequencing results of CRC and paired normal tissues showed that LINC01559 was expressed at low levels in CRC tissues compared with paired normal tissues. qRT-PCR analyses of CRC tissues and cell lines also proved the downregulation of LINC01559 in CRC, and low LINC01559 expression in CRC patients predicted poor survival. Furthermore, we investigated the downstream mechanism of LINC01559. Combined with the results of rescue experiments, LINC01559 could regulate the miR-106b-5p/PTEN axis to influence the biological functions of CRC cells. Previous mechanistic investigations of LINC01559 showed that METTL3 could methylate the m6A sites of LINC01559 to affect the functions of CRC cells.

## Methods

### Clinical samples

Fresh colorectal cancer tissues and paired adjacent normal specimens were collected by surgical resection from the First Affiliated Hospital of Zhengzhou University from October 2016 to February 2017. The adjacent normal tissues were normal intestinal mucosa that were more than 5 cm away from the tumor sites. The patients with colorectal cancer had received neither chemotherapy nor radiotherapy prior to resection. Pathological diagnostics for colorectal cancer were determined by three pathologists. The tumor stage was determined according to the eighth edition of the International Union Against Cancer (UICC)/American Joint Committee on Cancer (AJCC) TNM classification 15. The clinical characteristics of the 41 CRC patients are presented in Table 1. The deadline of follow-up was May 29^th^ 2019. Then, to avoid RNA degradation in tissues, we collected another 30 pairs of CRC tissues and adjacent normal tissues from January 2019 to July 2019. Prior to the use of these clinical materials, written consent was obtained from all patients, and approval was obtained from the First Affiliated Hospital of Zhengzhou University Ethical Review Committees.

### Bioinformatic analysis

Four CRC tumor samples and 3 normal intestinal mucosal tissues were collected at the First Affiliated Hospital of Zhengzhou University and analysed by lncRNA sequencing at RiboBio Biotechnology (Guangzhou, China). The results shown in Excel were filtered under both *p* value <0.05 and *q* value <0.05 conditions. Then, to plot a heatmap through Morpheus (https://software.broadinstitute.org/morpheus/), we set the FPKM below the mean as weakly expressed lncRNAs, and the FPKM above the mean as highly expressed lncRNAs in tumors respectively compared with normal samples.

In our research, KEGG analysis provided information about the signalling pathways in CRC (Additional file 1: Figure S1) 16, 17. MEM (https://biit.cs.ut.ee/mem/index.cgi) was utilized to explore genes sharing a coexpression relationship with LINC01559 (Additional file 2: Table S1) 18. Then, the first 100 genes were collected and pathway enrichment analyses were performed by KEGG through Webgestalt (http://www.webgestalt.org/) (Additional file 3: Figure S2) 19. The data from GEPIA (http://gepia.cancer-pku.cn/index.html) were utilized to analyse the correlation between PTEN and key molecules in different signalling pathways (Additional file 4: Figure S3) 20. The selection of potential miRNAs was performed by DIANA tools-Tarbase v8 (http://www.microrna.gr/tarbase) and DIANA tools-LncBase Predicted v2 (www.microrna.gr/LncBase) 21, and then a Venn diagram (http://www.cmbi.ru.nl/cdd/biovenn/) was utilized to intersect the two datasets (Additional file 5: Table S2) 22. Furthermore, we obtained GSE49246, GSE115513, GSE41655, GSE108153, GSE110402 and GSE56350 from GEO (http://www.ncbi.nlm.nih.gov/geo/) 23. StarBase 3.0 (http://starbase.sysu.edu.cn/) was used to analyse the binding site sequences of miR-106b-5p on LINC01559 and miR-106b-5p on PTEN 24. Then, the potential methylated sites of LINC01559 and miR-106b-5p were explored by RMBase v2.0 (http://rna.sysu.edu.cn/rmbase/) and SRAMP (http://www.cuilab.cn/sramp) (Additional file 6: Table S3, Additional file 7: Figure S4, Additional file 17: File S1) 25.

### *In situ* hybridization (ISH), imaging and scoring

After the tissue was dewaxed, it was incubated with the prehybridization solution and subsequently hybridized with the LINC01559 and miR-106b-5p probes. Then, visualization of the staining was performed using DAB. CRC cells were fixed with 4% paraformaldehyde in PBS for 20 min at room temperature. According to the manufacturer's instructions, LINC01559 and miR-106b-5p probes were used with a Fluorescent *In situ* Hybridization Kit (RiboBio, Guangzhou, China) in HCT116 and SW480 cells. Finally, images were obtained using confocal laser scanning microscopy (ZEISS, Jena, Germany). The signal intensities of LINC01559 and miR-106b-5p expression were quantified by using the intensity measurement tools of the Image-Pro Plus software package (Media Cybernetics, Houston, America). For quantification of ISH staining, samples were scored in a blind manner, and a staining H-score was calculated for each section, where H-score =Σ (% of cells with 4×4) + (% of cells with 3×3) + (% of cells with 2×2) + (% of cells with 1×1), in which 0 =no staining, 1 =weak, 2 =moderate, 3 =intense and 4 =very intense staining 26.

### Tumor xenografts in animals and functional assays *in vivo*

A total of 1×10^6^ logarithmic phase HCT116 cells were injected subcutaneously into the left and right flanks of 4- to 6-week-old BALB/c-nu/nu athymic nude mice (N =10) acquired from Vital River Laboratory (Beijing, China). Then, the mice were randomized into two groups: the anti-LINC01559 group (anti-LINC01559), and the anti-lincRNA control group (anti-lincRNA Ctl). The mice were treated with si-LINC01559 (10 nmol) or its negative siRNA control (RiboBio) in 50 μL saline buffer via direct injection into the tumor site once every 3 days for 6 rounds 27. Tumor size was measured by a slide calliper, and tumor volume was calculated as follows: volume =(D×d2)/2, where D was the longest diameter and d was the shortest diameter. For testing *in vivo* proliferation, the subcutaneous tumors were diced into 1 mm^3^ cubes and implanted into the mesentery at the caecum terminus of the nude mice. Animals were kept until the end of the experiment (20 days). The subcutaneous tumor tissue was fixed with 4% neutral buffered formalin and paraffin-embedded. Subsequently, consecutive tissue sections were made for each block and stained with haematoxylin-eosin (H&E) and IHC to observe the tumors in organs under a microscope. All specimens were examined under a light microscope (Nikon, Japan). Villus height and crypt depth were measured using an image analysis system. The experiments were performed according to institutional guidelines and approved by the Institutional Animal Care and Use Committee of Southern Medical University.

### RNA fluorescence *in situ* hybridization (FISH)

RNA FISH assays were performed to observe LINC01559 location. SW480, LoVo and HCT116 cells were fixed with 4% formaldehyde for 10 min at room temperature and then permeabilized using 0.5% Triton X-100 for 30 min. Afterwards, the cells were washed 3 × for 5 min in PBS and then hybridized with cDNA probe labelled with fluorochrome Cy3 (green) (Shanghai GenePharma Co., Ltd, Shanghai, China) (Fig. 6e, Additional file 10: Figure S5).

### Luciferase reporter assay

Wild-type LINC01559 with potential miR-106b-5p binding sites and a mutant of each site were generated and fused to the luciferase reporter vector psiCHECK-2 (Promega, Madison, WI, USA). The full-length wild-type (WT) 3' untranslated region (UTR) containing the predicted miR-106b-5p targeting site and the mutant-type (MT) 3'-UTR with a mutated miR-106b-5p binding site were amplified and cloned into the psiCHECK-2 vector. SW480 cells were placed on a 24-well plate and grown to 80% confluence. Cells were then cotransfected with luciferase plasmids and miR-106b-5p or control miRNA. After 48 h of transfection, firefly and Renilla luciferase activities were measured with a dual-luciferase reporter assay system (Promega). The same method was used to investigate the miR-106b-5p target sites in PTEN.

### RNA-immunoprecipitation assay (RIP) and RIP-sequencing (RIP-seq)

A Magna RIP RNA-Binding Protein Immunoprecipitation Kit (Millipore, USA) was used according to the manufacturer's instructions. Briefly, cells were collected and lysed with RIP lysis buffer. Then, magnetic beads coated with 5 μg of specific antibodies against mouse immunoglobulin G (17-700, Millipore) or METTL3 (Proteintech, Wuhan, China) were incubated with prepared cell lysates with rotation overnight at 4 °C. Then, the complexes were washed 6 times, and the precipitate was digested with Proteinase K buffer. Then, RNA was extracted by phenol-chloroform RNA extraction methods. Finally, library preparation was performed for RNA samples using the Illumina TruSeq Stranded mRNA Sample Prep Kit. No mRNA or rRNA depletion steps were performed. Libraries were sequenced by 50 bp paired-end sequencing.

### M6A RNA immunoprecipitation (MeRIP) assay

MeRIP was performed using the Magna MeRIP m6A Kit (Qiagen, Germany) according to the manufacturer's instructions. Briefly, 3 μg of anti-m6A antibody (Synaptic Systems, Goettingen, Germany) was conjugated to protein A/G magnetic beads overnight at 4 °C. Then, the antibody-conjugated beads were incubated with the antibody in IP buffer with RNase inhibitor and protease inhibitor. The interacting RNAs were isolated and detected by qRT-PCR.

### Statistics

All statistical analyses were conducted with SPSS version 18.0 (MT, USA) and GraphPad Prism 5.0 software (CA, USA). Data are expressed as the mean ± SEM. Two group pairs were compared by Student's t test. Pearson's coefficient was used to assess the correlations between variables. Survival data were obtained by the Kaplan-Meier method, with significance assessed by the log-rank test. The details of the analysis of these survival data are shown in the Supplemental Methods and Materials. The associations of LINC01559 expression and clinicopathologic variables were assessed by the chi-squared test or Fisher's exact test. Median LINC01559 expression levels were the cut-off points for determining high and low expression. *P* <0.05 was considered significant. Other methods are summarized in the supplemental information.

## Results

### LINC01559 is expressed at low levels in CRC, and the downregulation of LINC01559 is associated with poor prognosis

**Figure 2:**
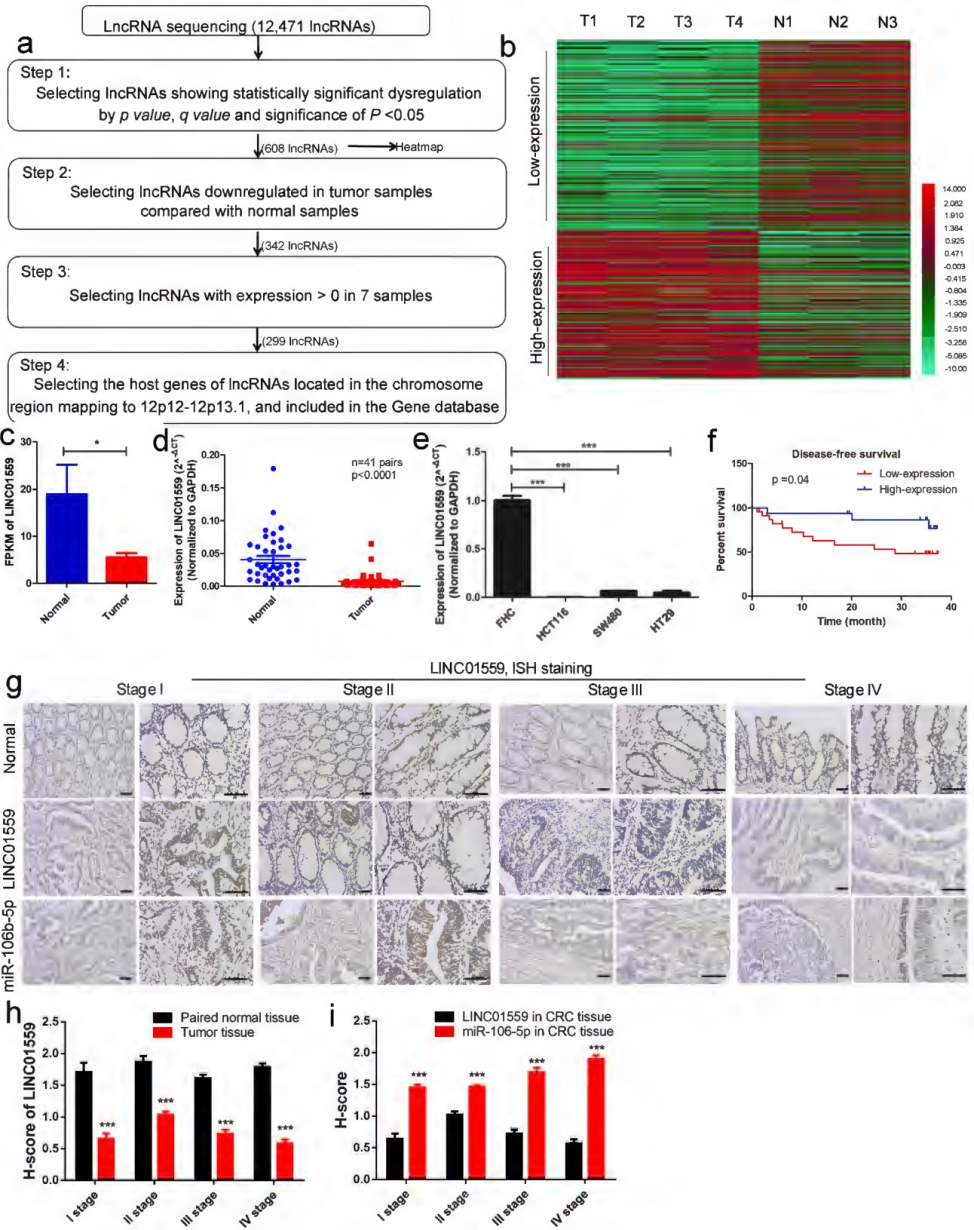
** LINC01559 is expressed at low levels in CRC, and the downregulation of LINC01559 is associated with poor prognosis. (a)** Flowchart illustrating the screening criteria of potential regulatory lncRNAs enriched in CRCs (*p* value <0.05, *q* value <0.05; T1, T2, T3 and T4 indicate tumor samples; N1, N2 and N3 indicate normal intestinal mucosa samples). **(b)** Clustered heatmap showing the dysregulated expression of lncRNAs after selection at Step 4. (Low expression means weakly expressed lincRNAs in tumor tissues; high expression means highly expressed lincRNAs in tumor tissues) **(c)** LINC01559 expression by lncRNA sequencing of 4 CRC tumor tissues (Tumor) and 3 adjacent normal tissues (Normal). **(d)** qRT-PCR analysis of LINC01559 expression in CRC tissues from patients (T, n =41) and paired normal tissues (N, n =41). **(e)** qRT-PCR analysis of LINC01559 expression in CRC cells (HCT116, SW480 and HT29) compared with the normal intestinal mucosa cells (FHC). **(f)** Kaplan-Meier plots of disease-free survival according to different LINC01559 expression groups (n =41, *P* =0.04). **(g)** and** (h)** ISH staining for LINC01559 or miR-106b-5p expression levels in paired normal tissue or CRC tissue, and H-score for ISH staining at different TNM stages (scale bar = 100 µm, scale bar =50 µm; Stage I n =1, Stage II n =2, Stage III stage n =3, Stage IV stage n =2; three images were obtained for each sample). The data in **(b)**, **(c)**, **(d)**, **(e), (f)**,** (h)** and** (i)** were analysed by Student's t test and are presented as the mean ± SD (^*^P <0.05, ^**^P <0.01, ^***^P <0.001).

Genomic copy number aberrations are believed to be an important driver of tumorigenesis 28, 29. The 12p12-12p13.1 region to which p27Kipl maps is of interest in relation to cancer, including CRC 11, 30. Thus, we investigated the dynamics of lncRNA alterations through lncRNA sequencing of 4 CRC tissue samples and 3 normal intestinal mucosa samples. Within these genomic loci, LINC01559 met the four following requirements: Step 1: lncRNAs showing significant dysregulation by by *p* value, *q* value and significance of *P* <0.05; Step 2: lncRNAs downregulated in tumor samples compared with normal samples by heatmap; Step 3: lncRNAs with expression >0 in 7 samples; Step 4: the host genes of lncRNAs located in the chromosomal region 12p12-12p13.1 and included in the Gene database (Fig. 2a, *P* <0.05). The clustered heatmap shows that 164 lncRNAs were identified after selection at Step 4 (Fig. 2b).

Next, four fresh cancer tissues and three adjacent normal tissues from patients were collected. The lncRNA expression profiles, determined by lncRNA sequencing, showed that the FPKM of LINC01559 was below the mean, indicating low expression compared with normal tissues (Fig. 2c, *P* <0.05). To verify the reliability of the RNA sequencing data, the RNA expression level of LINC01559 was detected by qRT-PCR in 41 pairs of tissues (Fig. 2d, *P* <0.0001) and 4 different cell lines, FHC, HCT116, SW480 and HT29 (Fig. 2e, *P* <0.001). The results revealed that LINC01559 was downregulated in CRC tissues and CRC cells compared with the normal group. To further characterize the association between LINC01559 expression and the outcome of CRC, patients were stratified according to the expression level of LINC01559. The Kaplan-Meier curves showed that a low expression level (below the median) of LINC01559 was associated with shorter disease-free survival in CRC patients (Fig. 2f, *P* =0.04). Then, we performed ISH staining to analyse LINC01559 expression and distribution in 10 pairs of CRC tissues and adjacent normal tissues from CRC patients with different TNM stages (Fig. 2g). Combined with analyses of the H-score, the results demonstrated that LINC01559 expression was lower in CRC tissues with different degrees of malignancy than in normal tissues (Fig. 2h, *P* <0.001).

A total of 41 patients, 16 males and 25 females, were examined. Their clinicopathological features are shown in Table 1. We selected the quartile expression of LINC01559 of 41 patients as the ideal cut-off value. Then, the CRC patients were divided into a high-expression level group (25 cases) and a low-expression level group (16 cases). The chi-squared tests showed that the different expression levels of LINC01559 were independently associated with TNM stage (*P* <0.05), lymphatic metastasis (*P* <0.05) and distant metastasis (*P* <0.01). Age, sex, tumor location, degree of differentiation, neural infiltration and vascular invasion were not significantly different between the LINC01559 expression groups (*P* >0.05). In conclusion, LINC01559 is expressed at low levels in CRC, and downregulated LINC01559 expression leads to poor prognosis, which indicates that LINC01559 is a tumor suppressor marker in CRC.

### Downregulated LINC01559 enhances CRC cell proliferation and metastasis *in vitro*

**Figure 3:**
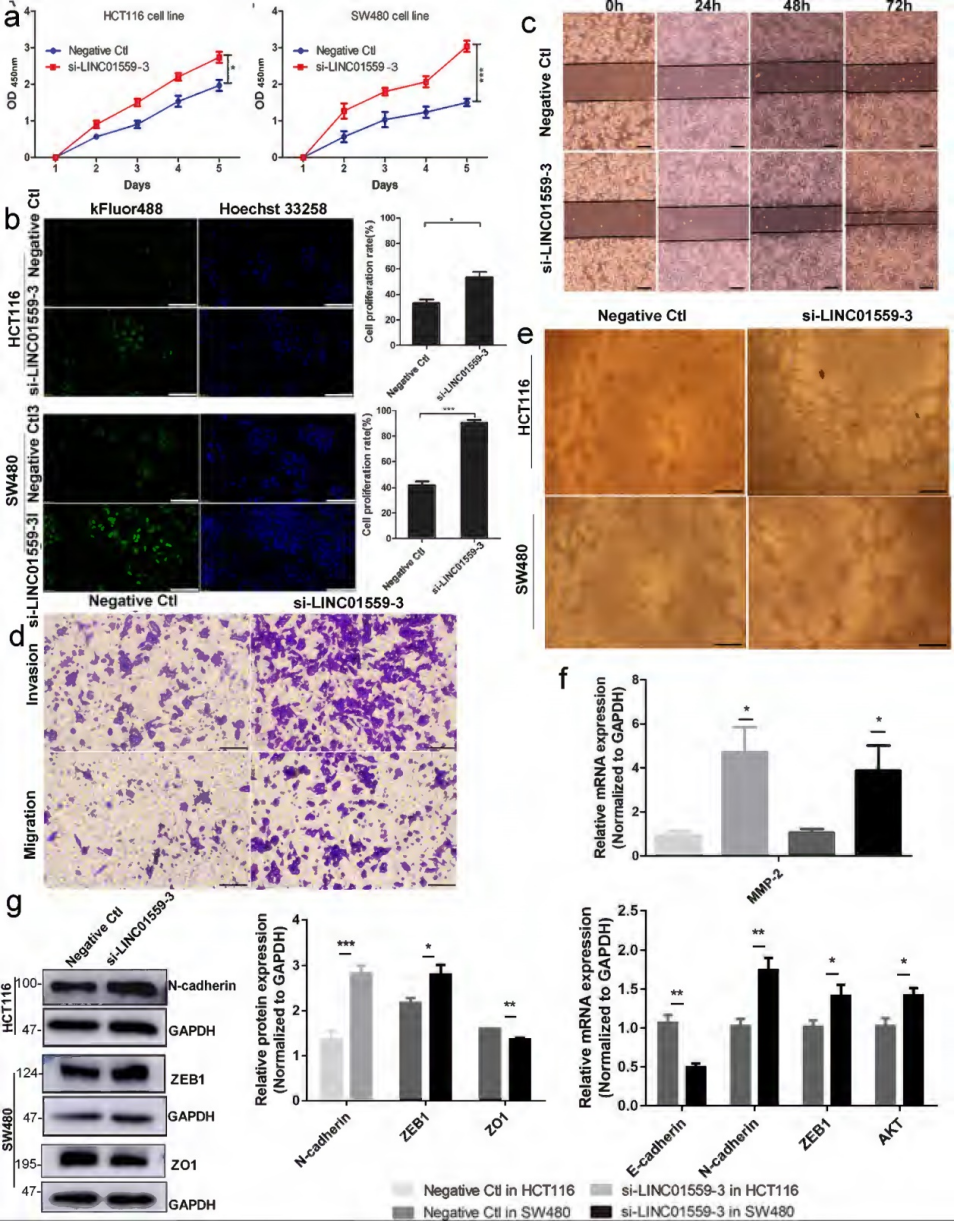
**Downregulated LINC01559 enhances CRC cell proliferation and metastasis *in vitro*.** HCT116 and SW480 cells were transfected with si-LINC01559-3 or the negative control vector (Negative Ctl). Cell proliferation was determined with a CCK-8 assay **(a)** and EdU staining (scale bar =50 µm) **(b)** Cell metastasis was detected through a wound healing assay (scale bar =100 µm) **(c)** and a Transwell assay (scale bar =50 µm) **(d)**. Representative images of the tube formation assay show the vascularization capacity of CRC cells **(e)** The results of differential gene expression with qRT-PCR **(f)** and WB (normalized to GAPDH) **(g)** The data in **(a)**, **(b)**, **(f)** and **(g)** were analysed by Student's t test and are presented as the mean ± SD (^*^P <0.05, ^**^P <0.01, ^***^P <0.001).

A qRT-PCR assay was utilized to estimate LINC01559 expression in HCT116 and SW480 cells. Then, we chose si-LINC01559-3 as a representative siRNA specific to LINC01559 because of its strong knockdown efficiency (Additional file 11: Figure S6). To assess the functions of LINC01559 in CRC cells, tumor cell proliferation was evaluated by CCK-8 and EdU assays. The results showed that silencing LINC01559 expression in HCT116 and SW480 cells considerably increased tumor proliferation (Fig. 3a, 3b). Furthermore, we used a wound healing assay to investigate whether LINC01559 was involved in the metastasis of tumor cells. The results showed that silencing LINC01559 expression in HCT116 and SW480 cells considerably induced their migration. The wound healing and Transwell assays revealed that, unlike transfection with the negative control sequence, siRNA-mediated knockdown of LINC01559 increased the metastasis of HCT116 and SW480 cells (Fig. 3c, 3d). Then, a tube formation assay was utilized to estimate the vascular formation ability of tumor cells. The results revealed that transfection with si-LINC01559-3 markedly induced vascularization in HCT116 and SW480 cells (Fig. 3e). To investigate the potential mechanism of LINC01559, WB and qRT-PCR assays demonstrated that silencing LINC01559 upregulated the RNA expression of MMP2 (*P* <0.05) in the two cell lines, N-cadherin (*P* <0.01), ZEB1 (*P* <0.05) and AKT (*P* <0.05) in SW480 cells but downregulated that of E-cadherin (*P* <0.01) (Fig. 3f). At the protein level, the WB analysis showed that LINC01559 silencing could improve the expression of N-cadherin (*P* <0.01) in HCT116 cells and ZEB1 (*P* <0.05) in SW480 cells but decrease that of ZO1 (*P* <0.001) (Fig. 3g). However, silencing LINC01559 did not obviously downregulate the expression of E-cadherin at the protein level (Additional file 12: Figure S7). Overall, silencing LINC01559 restricted this inhibitory effect on CRC cell proliferation and metastasis* in vitro*.

### Downregulated LINC01559 promotes CRC progression *in vivo*

**Figure 4:**
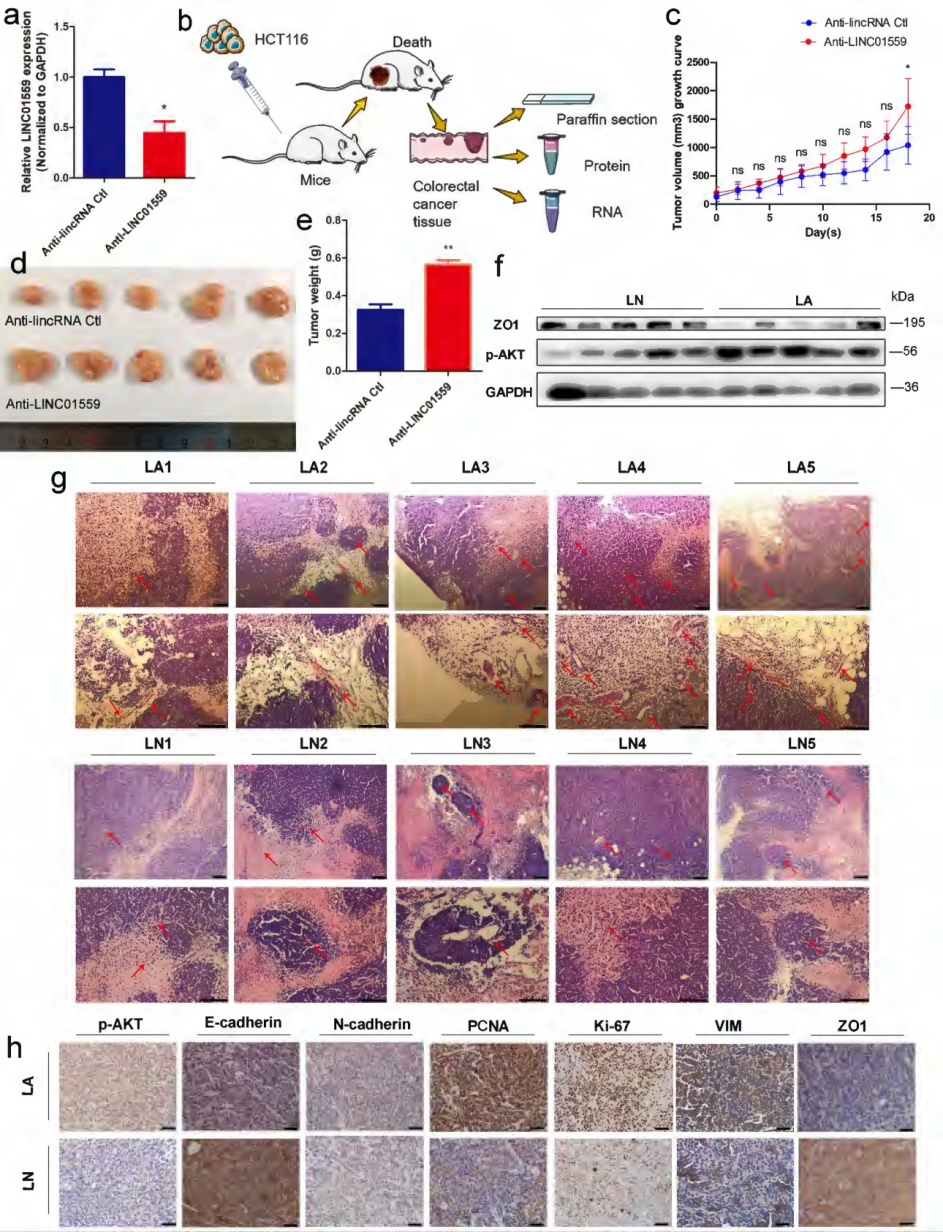
** Downregulated LINC01559 enhances CRC progression *in vivo*.** HCT116 cells were transfected with anti-LINC01559 or anti-LINC01559 control vector and then injected into nude mice. The subcutaneous tumor models were divided into two groups: the anti-LINC01559 negative control group (LN) and the anti-LINC01559 group (LA) (n =5 mice per group). qRT-PCR was used to examine the expression of LINC01559 to estimate the transfection efficiency (normalized to GAPDH)** (a)**. Schematic depiction showing the construction process of the CRC subcutaneous tumor model **(b)**. The tumor volume growth curves **(c)**, tumor size **(d)** and tumor weight **(e)** after implantation in different groups (n =5). WB analysis of ZO1 and p-AKT protein expression in LA compared with LN **(f)**. Representative images showing blood vessel distribution and density of tumor samples from different groups by H&E staining (scale bar =200 µm and 100 µm) **(g)**. Representative images showing the protein expression of p-AKT, E-cadherin, N-cadherin, PCNA, Ki-67, VIM and ZO1 from different tumor groups (scale bar =100 µm)** (h)**. The data in **(a)** and **(c)** were analysed by Student's t test and are presented as the mean ±SD (*P <0.05, **P <0.01, ***P <0.001, ^ns^: no significance).

**Figure 5:**
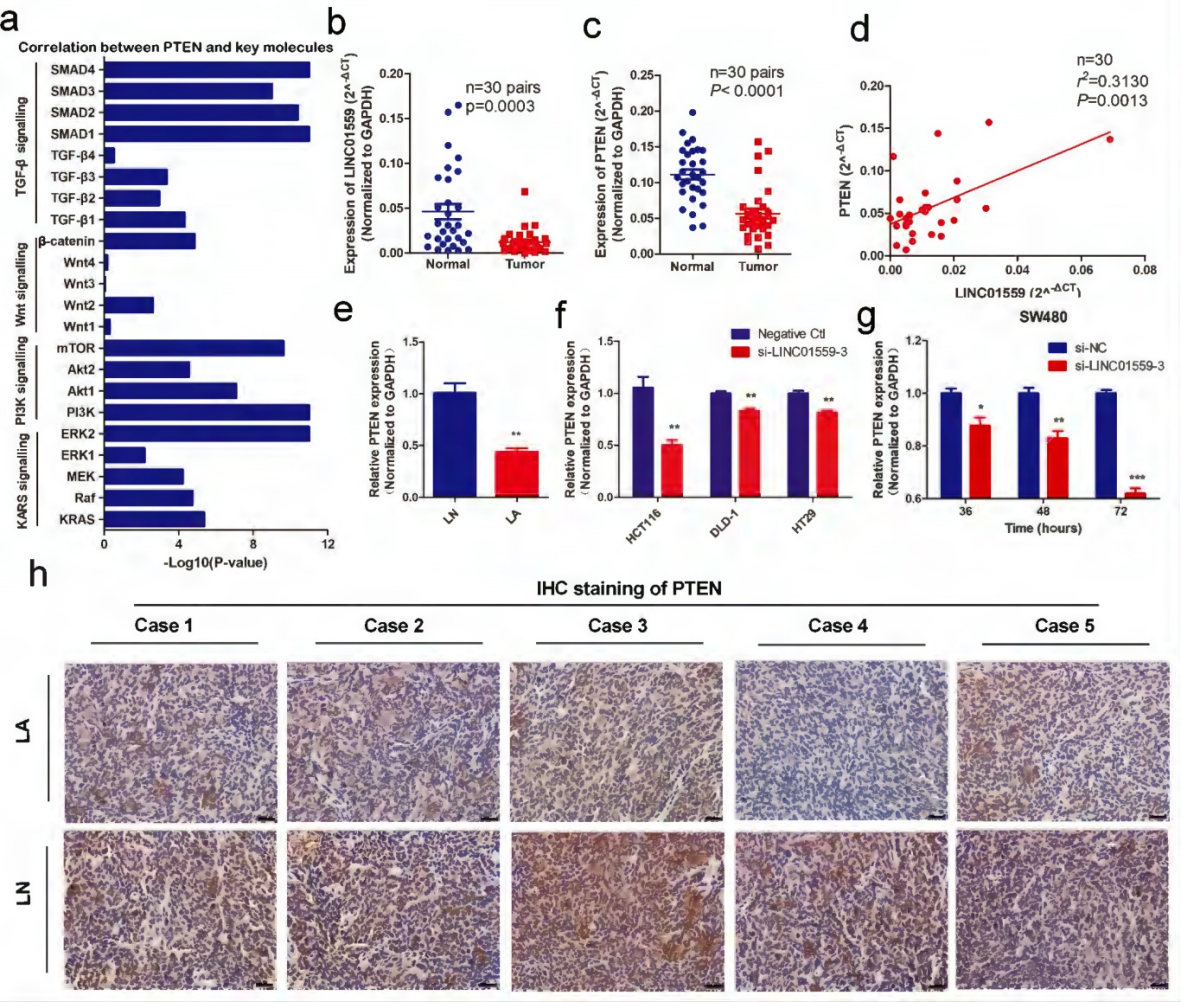
**PTEN is associated with LINC01559 and is expressed at low levels in CRC. (a)** The correlation between PTEN and key molecules in the signalling pathways of CRC. **(b, c)** LINC01559 and PTEN expression was detected by qRT-PCR in 30 pairs of colorectal cancer tissues (T) compared with adjacent normal tissues (N) (n =30, *P* <0.0001). **(d)** The correlation between PTEN mRNA expression and LINC01559 expression analysed in 30 pairs of colorectal cancer samples (n =30, *r*^2^ =0.31, *P* =0.0013) (normalized to GAPDH). **(e)** PTEN expression was detected by qRT-PCR in different tumor groups of subcutaneous tumor models (n =5) (normalized to GAPDH). **(f)** PTEN expression was detected by qRT-PCR in different cells transfected with si-LINC01559-3 or the negative control vector (Negative Ctl) (normalized to GAPDH). **(g)** qRT-PCR was used to explore PTEN expression in SW480 cells after 36, 48 or 72 h of transfection with si-LINC01559-3 or the negative control vector (Negative Ctl) (normalized to GAPDH). **(h)** Representative images showing the protein expression of PTEN in five mice bearing tumors from different groups (scale bar =100 µm). The data in **(a)**, **(b)**, **(c)**, **(d)**, **(e)** and **(g)** were analysed by Student's t test and are presented as the mean ± SD (^*^P <0.05, ^**^P <0.01, ^***^P <0.001).

To test the hypothesis that LINC01559 inhibits CRC progression, we transduced empty retroviral expression vectors and LINC01559 knockdown vectors with the anti-LINC01559 sequence into HCT116 cells. For comparison, the LINC01559 expression difference between the anti-LINC01559 negative control (anti-LINC01559 Ctl) and anti-LINC01559 in HCT116 cells was significant (*P* <0.05) (Fig. 4a). Then, the two groups of HCT116 cells were inoculated to nu athymic nude mice. After inoculation, the nude mice were randomly assigned into two groups of five mice each: the anti-LINC01559 negative control group (LN, treated with HCT116 cells transfected with empty vector) and anti-LINC01559 (LA, treated with HCT116 cells transfected with LINC01559 siRNA vector). Then, we observed tumor growth for 21 days and measured tumor volume and weight (Fig. 4b). The results showed that the tumor volume (*P* <0.05) and tumor weight (*P* <0.01) in the LA group were significantly higher than those in the LN group at the end of observation (Fig. 4c-e). The WB results demonstrated that the protein expression of ZO1 was also significantly downregulated in the LA group, while the protein expression of p-AKT was the opposite in the LA group (Fig. 4f). Furthermore, H&E staining was utilized to observe angiogenesis in the two groups. The results revealed that silencing LINC01559 considerably increased tumor vascularization (Fig. 4g). IHC staining demonstrated that the protein expression levels of p-AKT, N-cadherin, PCNA, Ki-67 and VIM were significantly increased in the LA group, while the results for E-cadherin, ZO1 and PTEN were the opposite (Fig. 4h, Fig. 5g). In summary, downregulated LINC01559 could enhance CRC cell progression *in vivo* by regulating CRC proliferation, angiogenesis and functional protein expression.

### PTEN is associated with LINC01559 and is expressed at low levels in CRC

Through analyses from MEM and Webgestalt, we found that the enriched pathways associated with tumor progression were mainly the p53 signalling pathway and PI3K-AKT signalling pathway (Additional file 2: Table S1, Additional file 3: Figure S2). Then, the results of preexperiment showed that, in SW480 cells transfected with si-LINC01559-3, the expression of AKT was upregulated (*P* <0.05) (Fig. 3f), while PTEN was significantly decreased (Fig. 5g). However, there was no significant difference in P53 expression between SW480 cells transfected with si-NC and si-LINC01559-3 (Additional file 13: Figure S8). Combined with previous research, PTEN is a tumor suppressor gene, and a downregulated PTEN expression status is associated with poor survival in CRC, which has been well characterized 31, 32. Considering that our noncoding RNA of interest, LINC01559, acts as a suppressor in CRC, we hypothesized that LINC01559 might influence the expression of PTEN. Next, we collected the key signalling pathways of CRC and analysed the correlation between PTEN and key molecules in these signalling pathways (Additional file 7: Table S4, Additional file 4: Figure S3). The results showed that PTEN was associated with several key molecules, including KRAS, Raf, MEK, ERK, PI3K, Akt, mTOR, Wnt, β-catenin, TGF-β and SMAD (Fig. 5a). To further confirm our prediction, another 30 pairs of CRC tissues were collected and the RNA expression of LINC01559 and PTEN was analysed by qRT-PCR. We found that the RNA levels of LINC01559 and PTEN were lower in 30 CRC tissues than in the paired normal tissues (*P* <0.0001, Fig. 5b, 5c). Furthermore, a correlation analysis was utilized to explore the correlation between LINC01559 and PTEN, revealing that LINC01559 expression is positively associated with PTEN in 30 pairs of CRC tissues (*P* =0.0031, Fig. 5d). Then, we used qRT-PCR to detect PTEN expression in a mouse model (Fig. 5e, *P* <0.01). We also analysed PTEN expression in CRC cell lines, including HCT116 (*P* <0.01), DLD-1 (*P* <0.01), and HT29 (*P* <0.01), which showed that downregulated LINC01559 could reduce PTEN expression at the RNA level (Fig. 5f). Moreover, PTEN expression at the RNA level was observed at 36 h (*P* <0.05), 48 h (*P* <0.01) and 72 h (*P* <0.001) in SW480 cells transfected with si-LINC01559-3 or the negative control vector. The results showed that the transfection efficiency of si-LINC01559-3 increased over time (Fig. 5g). The results of IHC staining *in vivo* demonstrated that the protein expression of PTEN was significantly decreased in the LA group (Fig. 5h). Thus, our results indicated that PTEN might be an anti-oncogene in CRC and could be regulated by LINC01559.

### MiR-106b-5p is associated with LINC01559 in CRC

**Figure 6:**
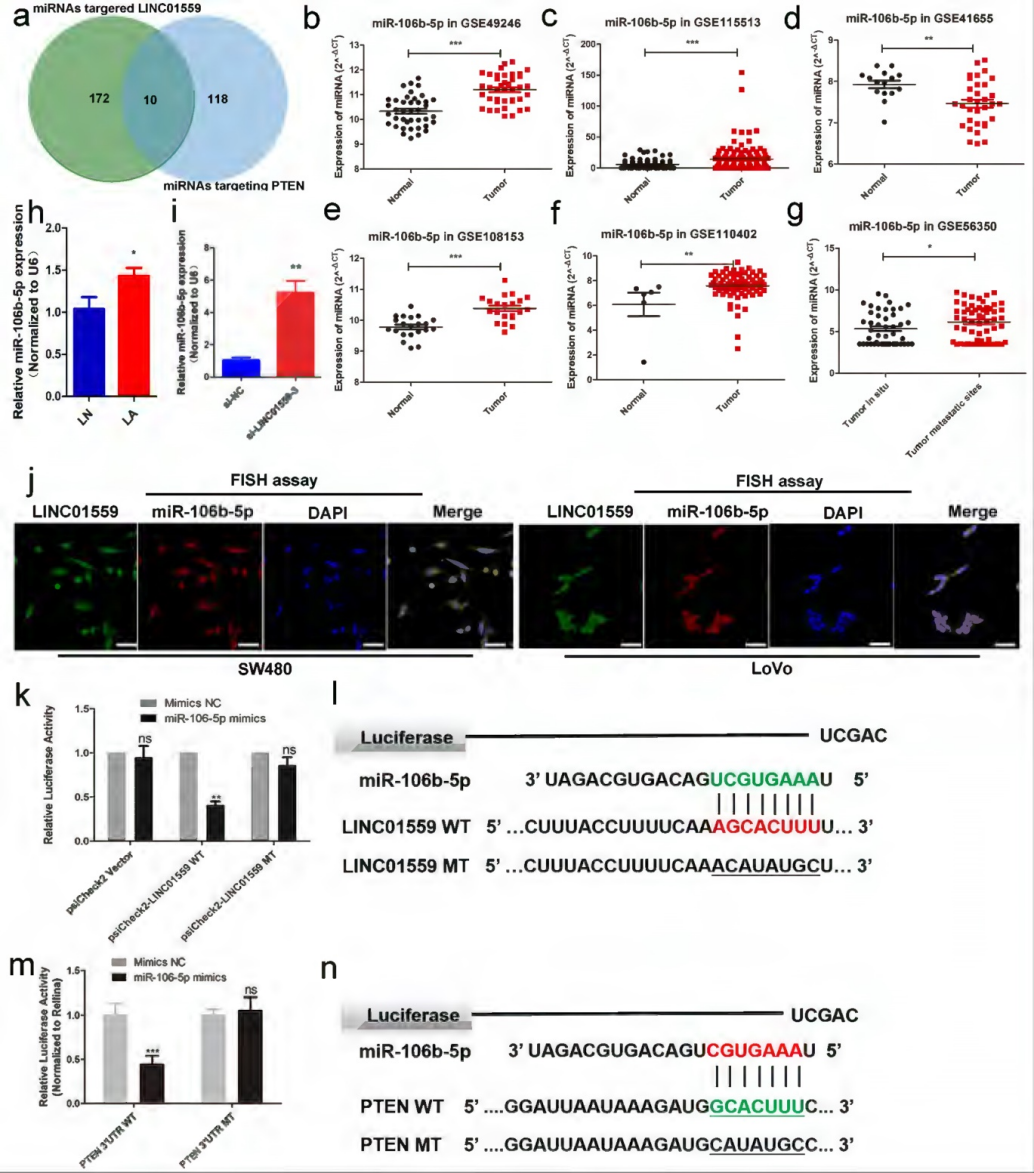
**miR-106b-5p is associated with LINC01559 in CRC. (a)** Venn diagram showing the shared miRNAs between miRNAs targeting LINC01559 and miRNAs targeting PTEN. **(b-g)** MiR-106b-5p expression was analysed in the GEO datasets GSE49246, GSE115513, GSE41655, GSE108153, GSE110402 and GSE56350. **(h)** MiR-106b-5p expression was detected by qRT-PCR in different groups of subcutaneous tumor models in nude mice (n =5). **(i)** MiR-106b-5p expression was measured by qRT-PCR in HCT116 cells transfected with si-LINC01559-3 or the negative control vector (Negative Ctl). **(j)** FISH assays were performed to determine the nuclear-cytoplasmic fractionation of LINC01559 and miR-10b-5p in SW480 and LoVo cells (green: LINC01559, red: miR-106b-5p, blue: DAPI, purple: merge, scale bar =50 µm). **(k)** A luciferase reporter assay was performed to detect the interaction between LINC01559 and miR-106b-5p. **(l)** The binding site sequences on LINC01559 for miR-106b-5p were obtained from StarBase 3.0 and mutated to complementary sequences. **(m)** A luciferase reporter assay was performed to detect the interaction between miR-106b-5p and PTEN (scale bar =50 µm). **(n)** The binding site sequences on miR-106b-5p for PTEN were obtained from StarBase 3.0 and were mutated to complementary sequences. The data in **(b)**, **(c)**, **(d)**, **(f)**, **(g)**, **(h)**, **(k)** and **(m)** were analysed by Student's t test and are presented as the mean ± SD (^*^P <0.05, ^**^P <0.01, ^***^P <0.001).

To investigate the mechanism by which LINC01559 regulates PTEN, we analysed the potential miRNAs targeted by LINC01559 via DIANA tools - Tarbase v8, and 172 miRNAs were identified. Then, we characterized 118 miRNAs containing complementary sequences with PTEN via DIANA tools - LncBase Predicted v2. Through a Venn diagram, we selected a total of 10 shared miRNAs for further study (Fig. 6a, Additional file 5: Table S2). Then, analysing miRNA expression in CRC via a GEO dataset (GSE49246), we found that only hsa-miR-17-5p, hsa-miR-20a-5p, hsa-miR-20b-3p and hsa-miR-106b-5p were significantly increased in CRC tumor tissues, while the other miRNAs showed no significant differences. Then, we further analysed the difference in miRNA expression between CRC tissues and normal tissues through other GEO datasets, including GSE115513, GSE41655, GSE108153 and GSE110402. Furthermore, GSE56350 from the GEO database provided RNA expression results in tumor tissues *in situ* and metastatic tumor tissues. The results showed that miR-106b-5p and miR-17-5p were dramatically highly expressed in tumor tissues compared with normal tissues. Moreover, the expression of the two miRNAs was higher in metastatic tumor tissues than in tumor tissues *in situ* (Fig. 6b-g, Additional file 14: Figure S9). Based on these associative data and previous relevant studies, we explored the role of miR-106-5p in the regulation of PTEN by LINC01559 33, 34. Confirming our previous findings, miR-106-5p was notably elevated in CRC tissues compared with normal tissues (Fig. 6h). Moreover, the expression of miR-106-5p was also increased with the downregulation of LINC01559 (Fig. 6i). Then, a FISH assay was utilized to examine LINC01559 distribution in the nucleus and cytoplasm. The results showed that LINC01559 was expressed both in the nucleus and cytoplasm and was more highly distributed in the cytoplasm (Fig. 6j).

To further determine whether miR-106b-5p could bind to the predicted target sites in LINC01559, we constructed three groups of LINC01559 luciferase reporter vectors: a blank group (psiCHECK-2 vector), a wild-type group (psiCHECK-2-LINC01559 WT) and a mutant-type group (the putative binding sites for miR-106b-5p were mutated, psiCHECK-2-LINC01559 MT). As expected, cotransfection with the wild-type LINC01559 vector (psiCHECK-2-LINC01559 WT) but not the mutant LINC01559 vector (psiCHECK-2-LINC01559 MT) with miR-106b-5p mimics significantly reduced luciferase activities in HCT116 cells (*P* <0.01, Fig. 6k). We focused on the targets of miR-106b and found via a bioinformatics search in StarBase 3.0 that the 3'-UTR of LINC01559 contained a region matching the seed sequence of miR-106b-5p (Fig. 6l). To study the relationship between miR-106b-5p and PTEN, we constructed three groups of miR-106b-5p luciferase reporter vectors: a blank group (psiCHECK-2 vector), a wild-type group (psiCHECK-2-PTEN WT) and a mutant-type group (putative binding sites for PTEN were mutated, psiCHECK-2-PTEN MT). We found that cotransfection of PTEN of the wild-type vector (psiCHECK-2-PTEN WT) but not the mutant PTEN vector (psiCHECK-2--PTEN MT) with miR-106b-5p mimics, significantly reduced luciferase activities in HCT116 cells (*P* <0.01, Fig. 6m). We also found that the 3'-UTR of PTEN contained a region that matched the seed sequence of miR-106b-5p (Fig. 6n). Finally, we analysed LINC01559 and miR-106b-5p expression in CRC tissues from different TNM-stage patients and paired normal tissues through ISH. ISH detected that LINC01559 expression in CRC tissues was lower than that in normal tissues, while miR-106b-5p expression was higher in CRC tissues (Fig. 2g, 2i). Altogether, our results indicated that LINC01559 might act as a molecular sponge for miR-106b-5p and that miR-106b-5p could regulate PTEN.

### LINC01559 regulates the functions of CRC cells via miR-106b-5p to promote PTEN

**Figure 7:**
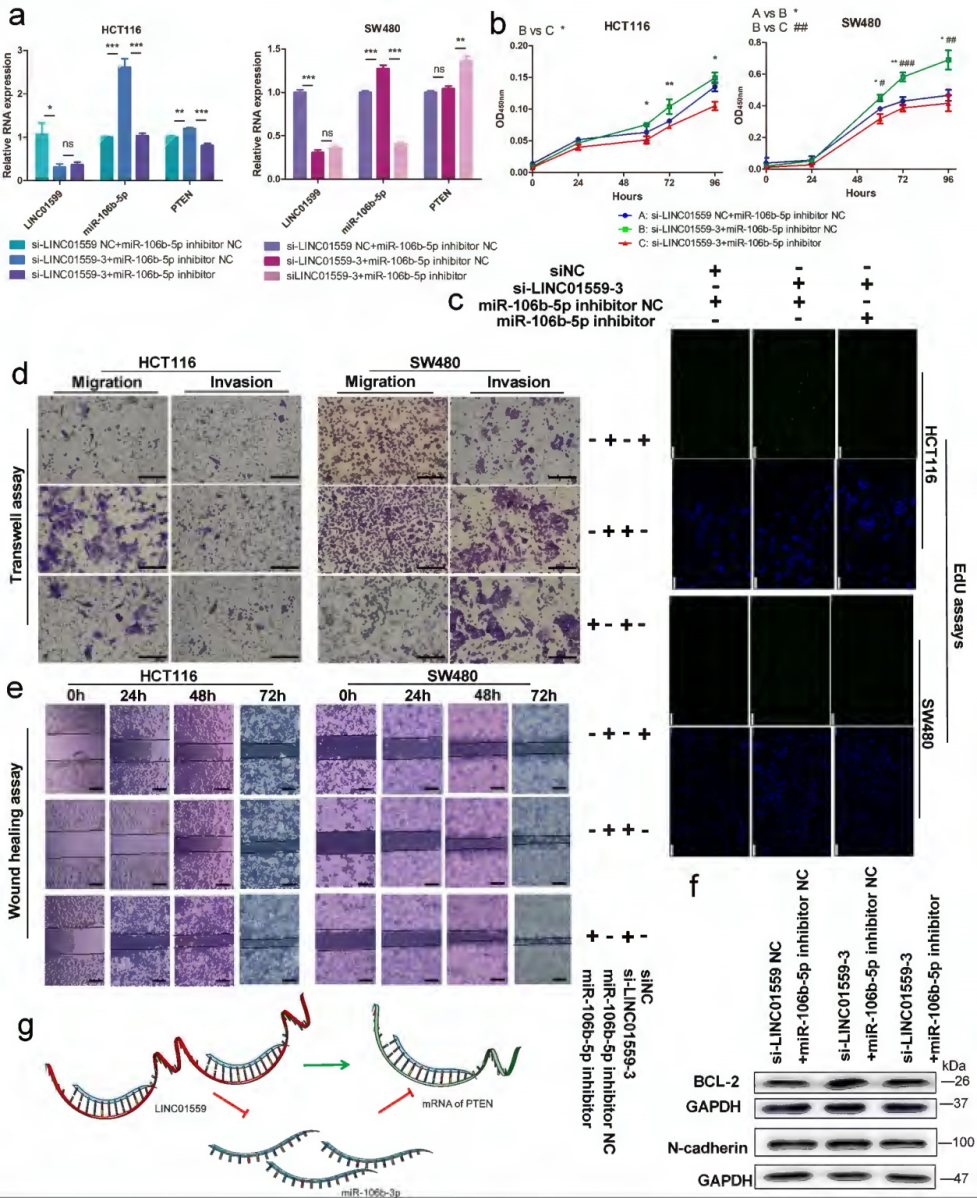
** LINC01559 regulates the functions of CRC cells via miR-106b-5p to promote PTEN.** HCT116 and SW480 cells were cotransfected with a siRNA against LINC01559 and a miR-106b-5p inhibitor. qRT-PCR was used to examine the expression of LINC01559, miR-106b-5p and PTEN after cotransfection (miR-106b-5p: normalized to U6, LINC01559 and PTEN: normalized to GAPDH) **(a)**. Cell proliferation was determined with a CCK-8 assay** (b)** and EdU staining (scale bar =100 µm) **(c)**. Cell migration and invasion were detected by Transwell assays (scale bar =50 µm) **(d)** and wound healing assays (scale bar =100 µm) **(e)**. The results of the WB test showed the protein expression levels of different genes **(f)**. Schematic representation of a model depicting that LINC01559 negatively regulated miR-106b-5p expression by interacting with PTEN **(g)**. In Figure 6b, A, blue: si-LINC01559 NC + miR-106b-5p inhibitor NC, B, green: si-LINC01559 + miR-106b-5p inhibitor NC, and C, red: si-LINC01559 + miR-106b-5p inhibitor. The data in **(a)** and **(b)** were analysed by Student's t test and are presented as the mean ± SD (Scale bar: 50 µm, ^*/#^ *P* <0.05, ^**/##^*P* <0.01, ^***/###^*P* <0.001, ^ns^: no significance).

To clarify the relationship between LINC01559 and PTEN with respect to miR-106b-5p regulation, HCT116 and SW480 cell lines were cotransfected with si-LINC01559 NC or si-LINC01559-3 and miR-106b-5p inhibitor NC or miR-106b-5p inhibitor. Then, HCT116 and SW480 cells were divided into three groups: si-LINC01559 NC + miR-106b-5p inhibitor NC, si-LINC01559-3 + miR-106b-5p inhibitor NC and si-LINC01559-3 + miR-106b-5p inhibitor. qRT-PCR assays showed that LINC01559 knockdown compromised the suppressive regulation of PTEN by miR-106b-5p, as indicated by the reduced levels in both HCT116 and SW480 cells (Fig. 7a). By CCK-8 assays and EdU assays, we found that LINC01559 knockdown promoted CRC cell proliferation, while simultaneous miR-106b-5p knockdown completely reversed the promotion of cell proliferation in both SW480 and HCT116 cells (Fig. 7b, 7c). Similarly, miR-106b-5p knockdown also reversed the promotion of CRC cell migration and invasion abilities using Transwell assays and wound healing assays (Fig. 7d, 7e). Downregulation of LINC01559 increased the protein expression of BCL-2 and N-cadherin in HCT116 cells, and this effect was counteracted by transfection with the miR-106b-5p inhibitor (Fig. 7f). However, si-LINC01559-3 did not downregulate the expression of E-cadherin (Additional file 15: Figure S10). Therefore, our results suggested a possible regulatory relationship in which LINC01559 negatively regulated miR-106b-5p expression by interacting with PTEN (Fig. 7g).

### METTL3 methylates LINC01559 to influence the function of CRC

**Figure 8:**
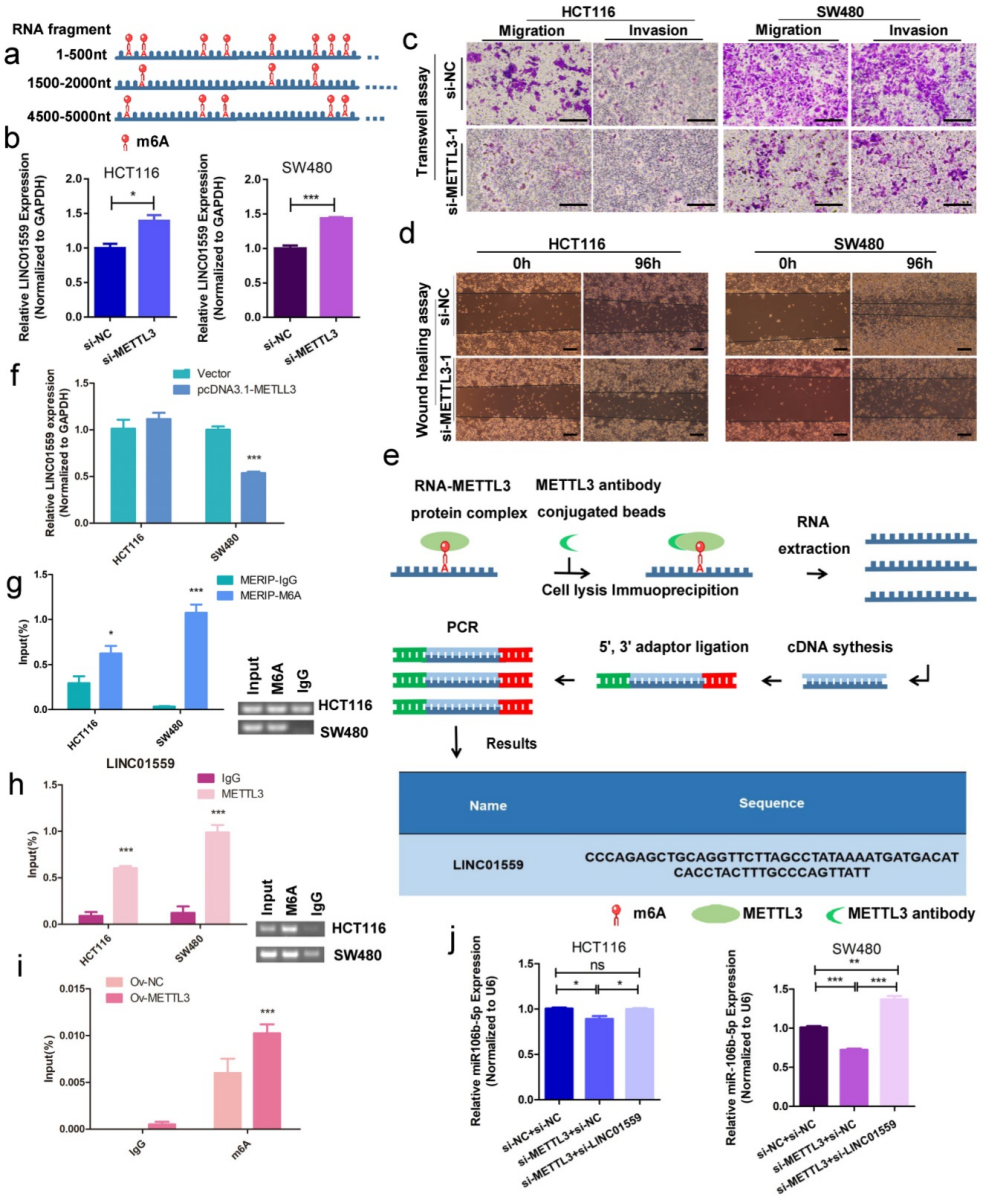
**METTL3 methylates LINC01559 to influence the function of CRC. (a)** Schematic representation of a model depicting m6A modification peaks in LINC01559. **(b)** Results of LINC01559 expression with qRT-PCR in HCT116 and SW480 cells transfected with si-METTL3 (normalized to GAPDH). Transwell assays (scale bar =50 µm) **(c)** and wound healing assays (scale bar =100 µm) **(d)** were utilized to determine the role of METTL3 in CRC**. (e)** The flow map of the MeRIP-qPCR process analysed by METTL3 protein antibody showed the possibility of METTL3 binding to LINC01559. **(f)** LINC01559 expression was detected by qRT-PCR in HCT116 and SW480 cells transfected with METTL3 overexpression vector (pcDNA3.1-METTL3) or negative control vector (vector). **(g)** MERIP assays and AGE assays were used to explore the enrichment of m6A modification in LINC01559. The percentage of the input in the SW480 group is shown. **(h)** RIP assays and AGE assays confirmed the binding between LINC01559 and METTL3 in HCT116 and SW480 cells. **(i)** MERIP assays detected the methylation sites of LINC01559 in SW480 cells transfected with OV-METTL3. **(j)** q-PCR assays were used to examine the expression of miR-106b-5p after cotransfection (miR-106b-5p: normalized to U6). The data in **(b)**, **(e)**, **(f), (g), (h), (i)** and **(j)** were analysed by Student's t test and are presented as the mean ± SD. (^*^P <0.05, ^**^P <0.01, ^***^P <0.001, ^ns^: no significance)

Bioinformatics analysis by RMBase v2.0 demonstrated that LINC01559 contains 17 m6A modification sites and m6A modification could influence LINC01559 regulation (Fig. 8a, Additional file 6: Table S3, Additional file 8: Figure S4). METTL3, as a key member of the m6A methyltransferase complex, has recently been reported to be highly expressed in metastatic CRC and associated with poor prognosis via diverse downstream genes 35. Then, on account of the observed strong knockdown or amplification efficiency, we chose si-METTL3-1 as a representative siRNA for METTL3 and OV-METTL3 in SW480 cells by qPCR assays (Additional file 16: Figure S11). Then, qRT-PCR results showed that downregulating METTL3 increased the expression of LINC01559 (Fig. 8b). Using Transwell assays and wound healing tests, we found that METTL3 knockdown inhibited CRC cell migration and invasion (Fig. 8c, 8d). To further explore whether the process of LINC01559 m6A methylation was influenced by METTL3, MeRIP-qPCR was performed, and the results indicated that METTL3 could bind to LINC01559 in SW480 cells (Fig. 8e). To explore whether METTL3 could modify LINC01559, cells with a stable increase in METTL3 were subjected to qRT-PCR. The results showed that overexpression of METTL3 inhibited the expression of LINC01559 in SW480 cells (*P* <0.001, Fig. 8f). Then, MERIP assays were used to explore the enrichment of m6A modification in LINC01559. Agarose gel electrophoresis (AGE) analysis following the MERIP assays was conducted to confirm the difference in RNA expression followed by immunoprecipitation with m6A antibody or IgG (control). The results revealed that the m6A modification in SW480 cells in the m6A group was approximately 32 times higher than that in the control group (normalized to the input, Fig. 8g). Accordingly, RIP assays and AGE were utilized to confirm the binding between LINC01559 and METTL3 in HCT116 and SW480 cells. The results showed that the complexes immunoprecipitated by the anti-METTL3 antibody contained overexpressed LINC01559 compared with the IgG group (normalized to the input, *P* <0.001, Fig. 8h). To further confirm the function of METTL3 in CRC cells, MERIP assays were used to detect the methylation sites of LINC01559 in SW480 cells transfected with OV-METTL3. The results showed that, compared with those in the group transfected with Ov-NC, m6A sites were upregulated by transfection with Ov-METTL3 in SW480 cells (normalized to the input, *P* <0.001, Fig. 8i).

To explore whether this activity, LINC01559 as a miR-106b-5p sponge, is mediated by METTL3, HCT116 and SW480 cells were cotransfected with si-METTL3 NC or si-METTL3 and si-LINC01559 NC or si-LINC01559. Then, HCT116 and SW480 cells were divided into three groups: si-METTL3 NC + si-LINC01559 NC, si-METTL3 + si-LINC01559 NC, and si-METTL3 + si-LINC01559. The results of the qRT-PCR assays showed that METTL3 downregulation compromised the inhibition of miR-106b-5p by LINC01559 in HCT116 and SW480 cells (Fig. 8j). Meanwhile, there were no potential methylated sites of miR-106b-5p according to the bioinformatics prediction by SRAMP (Additional file 17: File S1).

## Discussion

Previous studies have shown that lncRNA expression is altered in a variety of human cancer types and that lncRNA expression patterns may be associated with metastasis and disease prognosis 13, 23. Some reports also revealed that lncRNAs play an essential role in the tumorigenesis and progression of tumors, and lncRNAs might be involved in transcriptional regulation either as cis- or trans-acting elements and could negatively or positively influence gene expression 36. Furthermore, *in vivo* experiments also demonstrated that the expression of specific lncRNAs with oncogenic or anti-oncogenic features is closely linked with the ability to influence matrix invasion of cancer cells and tumor growth 37. Thus, research on the functions of lncRNAs in tumors is necessary to identify potential biomarkers in the prognosis and treatment of tumors. In our research, RNA-sequencing combined with qRT-PCR analysis revealed the downregulated expression pattern of LINC01559 in CRC tissues and cells, which was demonstrated to be associated with poor prognosis in CRC patients by clinical sample analysis. Our research highlighted the significant effects of LINC01559 during the complicated process of metastasis and proliferation through experiments *in vivo*. Consistent with our findings, silencing LINC01559 restored the migratory, invasive and proliferative abilities and affected a number of key molecules, including PTEN, N-cadherin, ZEB1 and MMP-2. However, LINC01559 downregulation could lower the RNA expression of E-cadherin but not the protein of E-cadherin. Indeed, E-cadherin serves as a widely acting suppressor of invasion and growth of epithelial cancers 38, while the result is the reverse in some kinds of cancer cells, including HCT116 cells 39. Therefore, the possible reason is that the expression of E-cadherin protein was high and could not be downregulated by si-LINC01559-3. The results on the whole predicted that LINC01559 played a depressed role in CRC progression. Increasing evidence could also be found in a previous study of rectal adenocarcinoma (READ) 40 and lung adenocarcinoma (LUAD) 41.While, LINC01559 was a positive predictor in a lncRNA risk prognostic nomogram of hepatocellular carcinoma (HCC) 42. Exosome-transferred LINC01559 from mesenchymal stem cells (MSCs) could be transferred into gastric cancer (GC) cells to promote GC cell progression 43. These pioneering studies implied that LINC01559 might have the different functions in tumor specificity.

Recent studies have proven that lncRNAs can affect diverse biological processes of cancers through the regulation of mRNA stability, RNA splicing, chromatin structure, and miRNA-mediated gene regulation by acting as miRNA sponges 7. To our knowledge, high levels of lncRNAs are located in the cytoplasm, and cytoplasmic lncRNAs function as modulators by interacting with miRNAs, such as by acting as competitive endogenous RNAs (ceRNAs) 44. The ceRNA network plays important roles in the occurrence and development of colorectal cancers, and most studies have demonstrated that lncRNAs and miRNAs are involved in ceRNA regulation 45. CeRNA crosstalk depends on the miRNA response elements (MREs) located on each transcript, which together form the foundation of these coregulatory interactions 46. In the present investigation, we demonstrated that miR-106b-5p could target MREs in LINC01559 through ceRNA interactions and could further influence the process of miR-106b-5p targeting PTEN.

We also explored the reason for the downregulated expression of LINC01559 in CRC cells. Recent studies have revealed alterations in the epigenetic regulation of ncRNAs via m6A methylation 47. The distribution and functions of m6A in lncRNAs are poorly understood. Previous studies have found that m6A is detected in lncRNAs and that lncRNAs could be substrates for adenosine methylation 48. Olfr29-ps1 relies mainly on the m6A-modified Olfr29-ps1/miR-214-3p/MyD88 regulatory pathway to modulate myeloid-derived suppressor cell (MDSC) immunosuppression and differentiation 49. Another study revealed that metastasis-associated lung adenocarcinoma transcript 1 (MALAT1) undergoes structural changes and localization due to m6A modifications and further regulates the interaction between RNAs and specific binding proteins 50. In addition, altering the m6A modification level of lncRNA-1281 can significantly affect let-7 levels, thereby influencing ESC differentiation. However, research on how m6A works on lncRNAs in CRC is limited. In our research, we found potential m6A sites of LINC01559, and METTL3 could modify these sites to regulate the function of LINC01559 in CRC, which is also one of the highlights of our research.

In this research, we first revealed that low LINC01559 expression could be an indicator of poor prognosis in patients with CRC. Furthermore, silencing LINC01559 significantly restored the proliferation ability of CRC cells. Research on the downstream molecular mechanism of LINC01559 demonstrated that the antioncogenic effect of LINC01559 is partly mediated through the inhibition of the activity of miR-106b-5p/PTEN. Notably, LINC01559 transcripts contain abundant m6A methylation sites, which provide potential targets for METTL3. Our data highlighted an innovative m6A-dependent RNA regulatory mechanism in epigenetics and indicated a promising biomarker of CRC for improving individualized treatment for CRC patients (Fig. 8).

## Limitations of the study

Although our study did not explore the whole possible pathway of the effect of LINC01559 in CRC, our research, based on adequate evidence, revealed that LINC01559/miR-106b-5p/PTEN is a promising axis for suppressing CRC progression. This research did not explore all possible methyltransferases of LINC01559. Meanwhile, MeRIP-qPCR assays confirmed that LINC01559 undergoes methylation modification and that upregulated or downregulated METTL3 could influence LINC01559 expression. In some way, the results revealed that METTL3 could promote the m6A methylation level of LINC01559 transcripts to interfere with the suppressor role of LINC01559 in CRC.

## Supplementary Material

Supplementary figures and table 3.

Supplementary table 1.

Supplementary table 2.

## Figures and Tables

**Figure 1:**
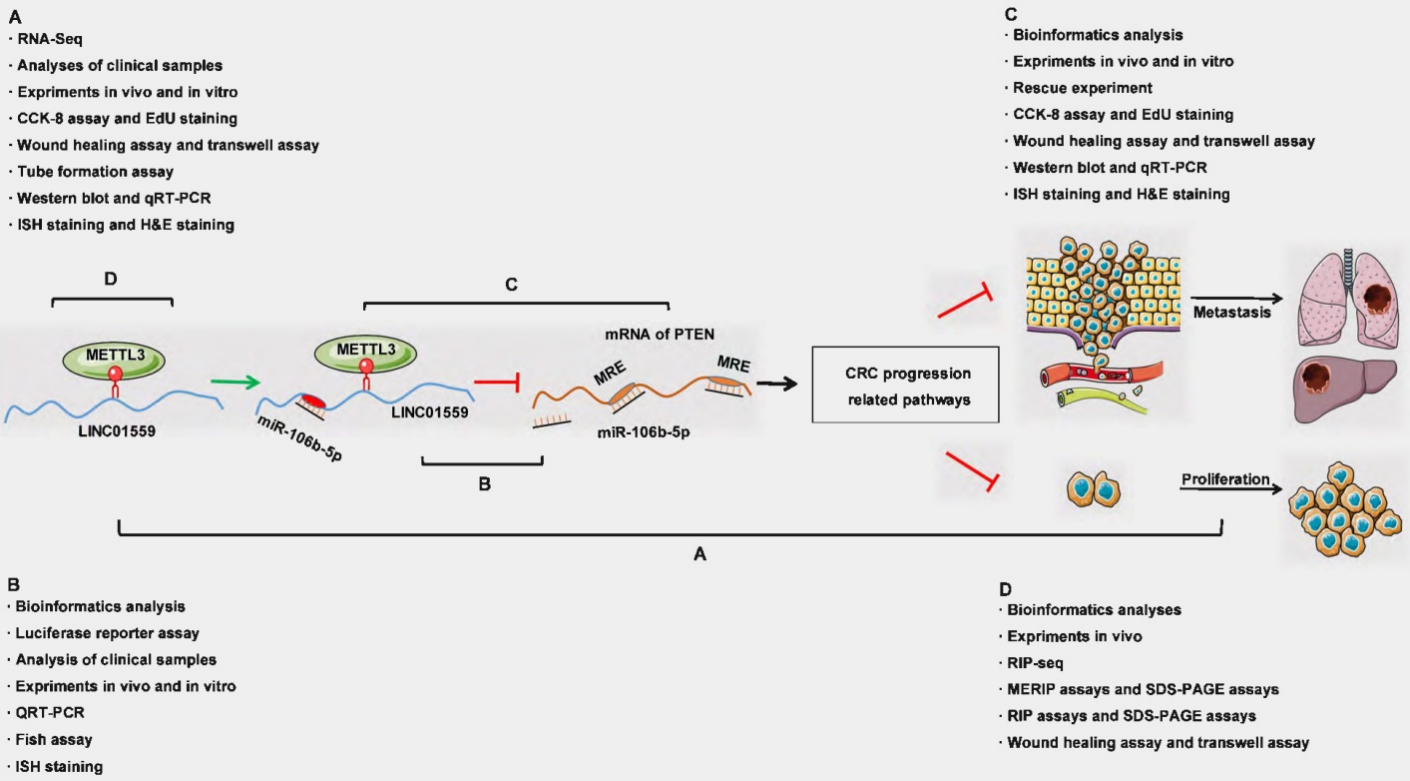
Schematic representation of a model depicting the major molecular mechanisms of the LINC01559/miR106b-5p/PTEN axis in CRC.

**Table 1:** Univariate analyses of the relationship between LINC01559 and clinicopathological parameters in patients with CRC

Characteristics	n	LINC01559		χ^2^	*P*
		Low	High		
**Age**				0.141	0.707
<60	19	11	8		
≥60	22	14	8		
**Gender**				2.169	0.141
Male	16	12	4		
Female	25	13	12		
**Tumor size (cm)**				0.137	0.412
<5	25	16	9		
≥5	7	5	2		
**Tumor location**				0.570	0.450
Colon	10	7	3		
Rectum	30	17	13		
**Histological differentiation**				2.575	0.109
Well/middle	15	7	8		
Poorly/undifferentiated	22	16	6		
**TNM stage**				9.471	0.002
I/II/III	20	17	3		
IV	21	8	13		
**Lymph node metastasis**				5.184	0.023
Yes	25	19	6		
No	16	6	9		
**Distant metastasis**				6.740	0.009
Yes	18	15	3		
No	23	10	13		
**Perineural invasion**				0.577	0.448
Yes	10	5	5		
No	25	16	9		
**Vascular invasion**				0.162	0.685
Yes	14	9	5		
No	26	15	11		

## Abbreviations

miRNAs: microRNAs
mRNA: messenger RNA
3′UTR: 3′ untranslated region
EMT: epithelial-to-mesenchymal transition
qRT-PCR: quantitative reverse transcription polymerase chain reaction
CRC: colorectal cancer
TRN: trial registration number
OS: overall survival
WT: wild-type
READ: rectal adenocarcinoma
HCC: hepatocellular carcinoma
RCC: renal cell carcinoma
LUAD: lung adenocarcinoma
m6A: N6-methyladenosine
METTL3: methyltransferase-like 3
FISH: RNA fluorescence in-situ hybridization
RIP: RNA immunoprecipitation
RIP-seq: RNA immunoprecipitation sequencing
MeRIP: m6A RNA immunoprecipitation
H&E: haematoxylin-eosin
OV-METTL3: overexpressed METTL3 group
iCCAs: intrahepatic cholangiocarcinoma
MDSC: myeloid-derived suppressor cells
MALAT1: metastasis-associated lung adenocarcinoma transcript 1
NC: negative control
LN: anti-LINC01559 negative control group
LA: anti-LINC01559 group
WB: western blot analysis
MRE: miRNA response elements
AGE: agarose gel electrophoresis
mRNAs: messenger RNAs
MSCs: mesenchymal stem cells
GC: gastric cancer
