# Supplementary material for: RNA methylation-mediated LINC01559 suppresses colorectal cancer progression by regulating the miR-106b-5p/PTEN axis
Source: Int J Biol Sci. 2022 Apr 24;18(7):3048–65. doi: 10.7150/ijbs.70630 (PMC9066122; doi:10.7150/ijbs.70630)
Supplement: Supplementary file 1 — Supplementary figures and table 3. [file ijbsv18p3048s1.pdf]

Supplemental Methods and Materials

Cell culture

Cell culture was adapted from a previous publication [29]. The human CRC cell lines (HCT116, SW480, FHC, HT29, DLD-1) were obtained from iCell Bioscience lnc (Shanghai, China) and authenticated by the STR before we used. All cells were cultured in DMEM (High Glucose) (Hyclone, Logan, Australia) with 10% fetal bovine serum (BI, Israel) under the condition with 37°C and 5% CO_2.

Quantitative real time polymerase chain reaction (qRT-PCR)

Total RNA was extracted from CRC cell lines and patient specimens using TRIzol reagent (Life Technologies) according to the manufacturer's manual. One microgram of total RNA was used as template for cDNA synthesis using a PrimeScript RT Reagent Kit with cDNA Eraser (Vazyme, Nanjing, China) and qRT-PCR was performed using SYBR Premix Ex Taq (Takala, Dalian, China). MiRNA expression was performed in triplicate using SYBR PrimeScriptTM miRNA RT-PCR Kit (Takara Biotech). All qRT-PCR assays was performed on an ABI 7900 system (Applied Biosystems, Foster City, CA, USA). Expression levels of genes or miRNA were normalized to that of the housekeeping gene glyceraldehyde 3-phosphate dehydrogenase (GAPDH). $2-\Delta\Delta Ct$ method was applied for calculation of relative levels of genes and miRNA expression. All primers are list in Additional file 9: Table S5 (Sunya Primer Synthesis Company, Zhengzhou, China).

Plasmids construction and cell transfection

The siRNA of LINC01559, miR-106b-5p, PTEN and METTL3 was designed and synthesized by RiboBio (Guangzhou, China). The knowdown effiency was analyzed through q-PCR, and marked one was chosen for further experiments. The details of siRNA sequences were showed in Additional file 7: Table S4. The overexpression vector of METTL3 was pcDNA3.1 (Invitrogen, ThermoFisher Scientific, Carlsbad, CA, USA). According to the manufacturer's instructions, lipofectamine 3000

(Invitrogen, ThermoFisher Scientific, Carlsbad, CA, USA) was used to transient siRNA and overexpression vector transfection.

Western blot analysis (WB)

Total proteins were prepared from CRC using RIPA buffer (50 mm Tris-HCl, 150 mm NaCl, 1 mm EDTA, 0.1% SDS, 1% Triton X-100, 0.1% sodium deoxycholate) with proteinase inhibitor cocktail (Solarbio, Beijing, China). The lysates were centrifuged at 12,000 rpm for 15 min at 4 °C and protein concentration was measured by BCA kit (Beyotime Biotechnology, Beijing, China). Equal quantities of protein were electrophoresed through a 10% sodium dodecyl sulfate/polyacrylamide gel and transferred to PVDF membranes (Millipore, Massachusetts, MA). The PVDF membranes were blocked with skim milk powder dissolved by TBST (5%) at room temperature for 1 h, and then incubated with primary antibodies at 4 °C overnight. The primary antibodies included anti-N-cadherin (No. 66219-1-Ig), anti-E-cadherin (No. 20874-1-AP), anti-ZEB1 (No. 21544-1-AP), anti-ZO-1 (No. 21773-1-AP) from Proteintech (Wuhan, China), and anti-p-AKT (No. 4060), and anti-BCL-2 (No. 15071) from Cell Signaling Technology (MA, USA). Secondary antibodies hybridized the membrane at room temperature for 1 h. Then the membranes were visualized by the chemiluminescence kit (Absin, Shanghai, China). WB strips were detected through Image-Pro Plus 6.0 and analyzed through Student's t test.

Wound healing assay

Cells were cultured in standard conditions until 80–90% confluence and treated with mitomycin C (10 μg/ml) during the wound healing assay. The cell migration was assessed by measuring the movement of cells into the acellular area created by a sterile insert. The wound closure was observed after 48 h.

Transwell assays

To assess the migration and invasiveness of HCT116 and SW480 cells, we used Transwell chambers (Corning, NY, USA). Briefly, 3×10^5 cells in serum-free medium

were placed in the upper chamber. Dulbecco's modified Eagle's medium (500 ml) supplemented with 10% fetal bovine serum was added to the lower chamber. After incubation in a humidified atmosphere containing 5% CO_2 at 37°C for 72 h, Giemsa staining of A375 cells that had migrated or invaded into the lower chamber was performed. Stained cells were photographed under an IX53 inverted microscope (NIKON, Tokyo, Japan), and the Image-Pro Plus software program (Media Cybernetics, Rockville, MD) was used to count the cells.

Cell proliferation assay

Cells were seeded in 96-well plates at 0.8–1×10^3 per well. Cell proliferation was evaluated using Cell Counting Kit-8 (Dojin Laboratories, Tokyo, Japan) according to manufacturer's instructions. We collected cell samples at 24h, 48h, 72h and 96h, respectively. Then 10 μl of CCK-8 solution was added to culture medium, and incubated for 2h. The absorbance at 450 nm wavelength was determined with a reference wavelength of 570 nm.

5-ethynyl-2′-deoxyuridine assay (EdU)

A total of 4×10^3 cells per well were seeded into 96-well plates and cultured overnight, and then washed with phosphate-buffered saline (PBS), fixed with 4% paraformaldehyde for 30 min, and incubated with 2 mg/ml glycine for 5 min. Based on the kFluor488-EdU (5-ethynyl-2′-deoxyuridine) manufacturer's instructions (Ribobio, Guangzhou, China), 200 μl of 1× Apollo dyeing solution was added to each well, followed by incubation at room temperature for 30 min. Next, 100 μl of 0.5% Triton X-100 was used to wash the cells two- to three-times (10 min per wash). Following staining with Hoechst 33342 at room temperature for 30 min in darkness and one or two washes with PBS, the cells were observed using a Micro system (ImageXpress, Downingtown, PA, U.S.A.). Five fields were randomly selected and photographed, and the number of EdU-positive cells was calculated.

Tube formation assay

Twenty-four-well plates were coated with 60 ml Matrigel (BD Biosciences, USA) at 37 °C for 1 h for gel formation. A total of 1×10^5 stably transfected cells in medium containing 10% FBS were plated into the pre-solidified Matrigel and started the process to form capillary tubes and networks once seeded on Matrigel. Six hours after incubation, plates were observed under microscope and photographed (Nikon, Japan). The numbers of branching points generating at least three tubules were counted.

The statistical analysis of survival data

The data of DFS and follow-up data (current status, survival) was obtain through telephone follow-up survey, periodic re-examination and readmission. CRC recurrence or death were regarded as terminal point event. The days of DFS were calculated from date of diagnosis to date of terminal point event occurrence. The expression of LINC01559 was detected through q-PCR assays in 41 pairs of CRC tissues and adjacent normal tissues. The high level of LINC01559 was designed as the expression above the median, and the low level was designed as the expression below the median. The outcome variable 1 represented CRC recurrence or death due to CRC and 0 represented censoring (loss of follow-up, endpoint inoccurrence or others). Survival data were obtained by the Kaplan-Meier method, with significance assessed by the log-rank test in SPSS.

Legends to Supplemental Material

Supplemental Methods and Materials

Additional file 1: Figure S1. Key signaling pathways in CRC from KEGG.

Additional file 2: Table S1. The first 100 genes genes sharing co-expression relationship with LINC01559.

Additional file 3: Figure S2. Pathway enrichment analyses in coexpression genes of LINC01559.

Additional file 4: Figure S3. The correction between PTEN and some key molecules in signaling pathways: KRAS**(a)**, Raf **(b)**, MEK **(c)**, ERK1 **(d)**, ERK2 **(e)**, PI3K **(f)**, Akt1 **(g),** Akt2 **(h)**, mTOR **(i)**, Wnt1 **(j)**, Wnt2 **(k)**, Wnt3 **(l)**, Wnt4 **(m)**, β-catenin **(n)**, TGF-β1 **(o)**, TGF-β2 **(p)**, TGF-β3 **(q)**, TGF-β4 **(r)**, SMAD1 **(s)**, SMAD2 **(t)**, SMAD3 **(u)** and SMAD4 **(v)**.

Additional file 5: Table S2. Selection of miRNAs: Sheet 1–miRNAs targeted by LINC01559; Sheet 2 – miRNAs targeting PTEN; Sheet 3 – shared miRNAs.

Additional file 6: Table S3. Prediction of the potential methylated sites of LINC01559 by RMBase v2.0.

Additional file 7: Table S4. The correction between PTEN and key molecules in signaling pathways of CRC.

Additional file 8: Figure S4. m6A motif result of GSM1339411 **(a)**, GSM1339429 **(b)**, GSM1339431 **(c)**, GSM1339441 **(d)**, GSM2203055 **(e)**, GSM2203056 **(f)**, GSM2203060 **(g)** and GSM2460345 **(h)**.

Additional file 9: Table S5. All primers and siRNA used in cytological experiments.

Additional file 10: Figure S5. FISH assays in HCT116 (scale bar: 50 μm).

Additional file 11: Figure S6. qRT-PCR assay was utilized to estimate the knockdown efficiency of si-LINC01559 in HCT116 and SW480 cells.

Additional file 12: Figure S7. WB assay was utilized to estimate the E-cadherin expression in HCT116 and SW480 cells transfected by si-LINC01559-3.

Additional file 13: Figure S8. P53 expression in SW480 tranfected with si-LINC01559-3 through q-PCR.

Additional file 14: Figure S9. Through the data from GSE49246, the analyses of expression difference between CRC tissues and paired normal tissues in ten selected shared miRNAs, including miR-20b-5p in GSE49246 **(a)**, miR-17-5p in GSE49246 **(b)**, miR-9-3p in GSE49246 **(c)**, miR-320a in GSE49246 **(d)**, miR-320b in GSE49246 **(e)**, miR-423-5p in GSE49246 **(f)**, and miR-20a-5p in GSE49246 **(g)**. We found that miR-17-5p, miR-20a-5p, miR-20b-3p and miR-106b-5p were significantly decreased in CRC tumors tissues, while the other miRNAs showed no significant differences. MiR-320c and miR-320d could not be included in GSE49246. Moreover, the data from GSE115513, GSE41655, GSE110402, GSE108153 and GSE56350 was used for further selection, the result included miR-20b-5p in GS115513 **(h)**, miR-17-5p in GSE 115513 **(i)**, miR-20a-5p in GSE115513 **(g)**, miR-20b-5p in GSE41655 **(k)**, miR-17-5p in GSE41655 **(l)**, miR-20a-5p in GSE41655 **(m)**, miR-20b-5p in GSE110402 **(n)**, miR-17-5p in GSE110402 **(o)**, miR-20a-5p in GSE110402 **(p)**, miR-20b-5p in GSE108153 **(q)**, miR-17-5p in GSE108153 **(r)**, miR-20a-5p in GSE108153 **(s)**, miR-20b-5p in GSE56350 **(t)**, miR-17-5p in GSE56350 **(u)**, and miR-20a-5p in GSE56350 **(v)**. The data were analyzed by students t-test and were presented as the mean ± SDs. (Red: higher expression of miRNAs in CRC tissues, Green: lower expression of miRNAs in CRC tissues, *$P < 0.05$, **$P < 0.01$, ***$P < 0.001$, ^{ns}: no significance)

Additional file 15: Figure S10. WB assay was utilized to estimate the E-cadherin expression in CRC cells transfected by si-LINC01559-3 or si-NC and miR-106b-5p inhibitor NC or miR-106b-5p inhibitor or miR-106b-5.

Additional file 16: Figure S11. qRT-PCR assay was utilized to estimate the over-expressed efficiency of OV-METTL3 in HCT116 and SW480 cells and si-METTL3 efficiency of SW480.

Additional file 17: File S1. Prediction of m6A sites on miR-106b-5p based on sequence-derived features.

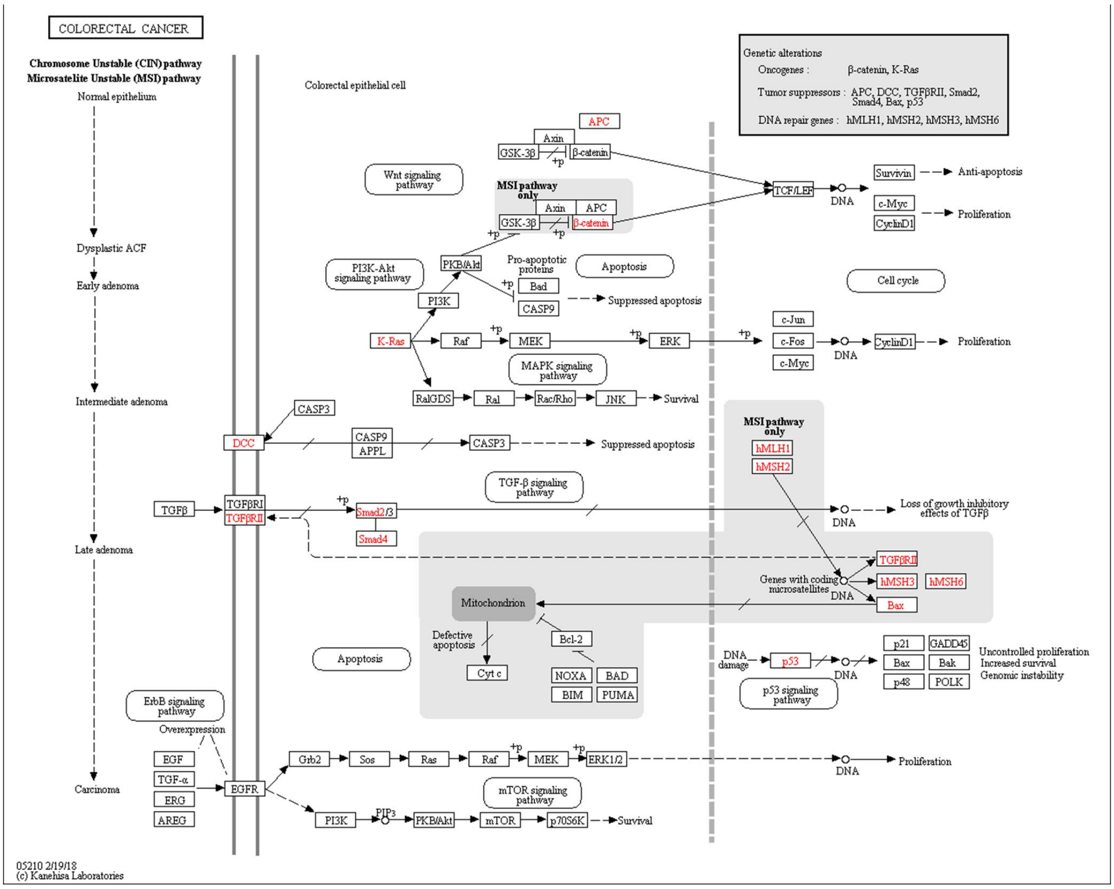

Figure S1

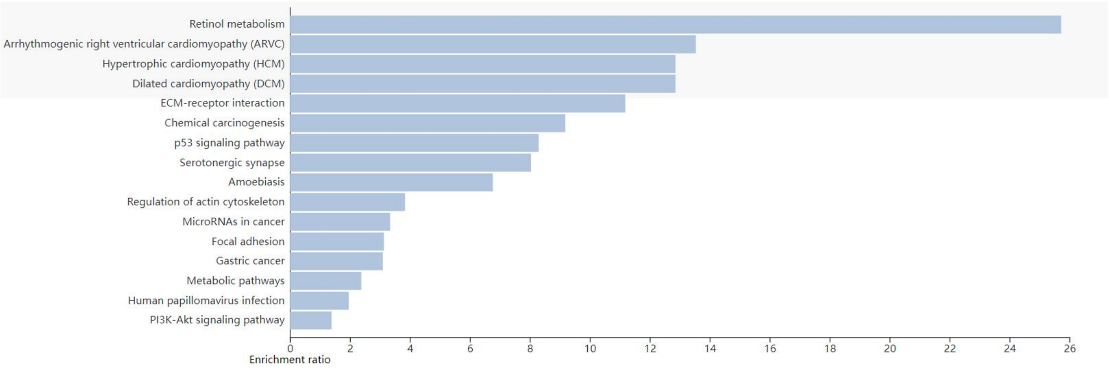

Figure S2

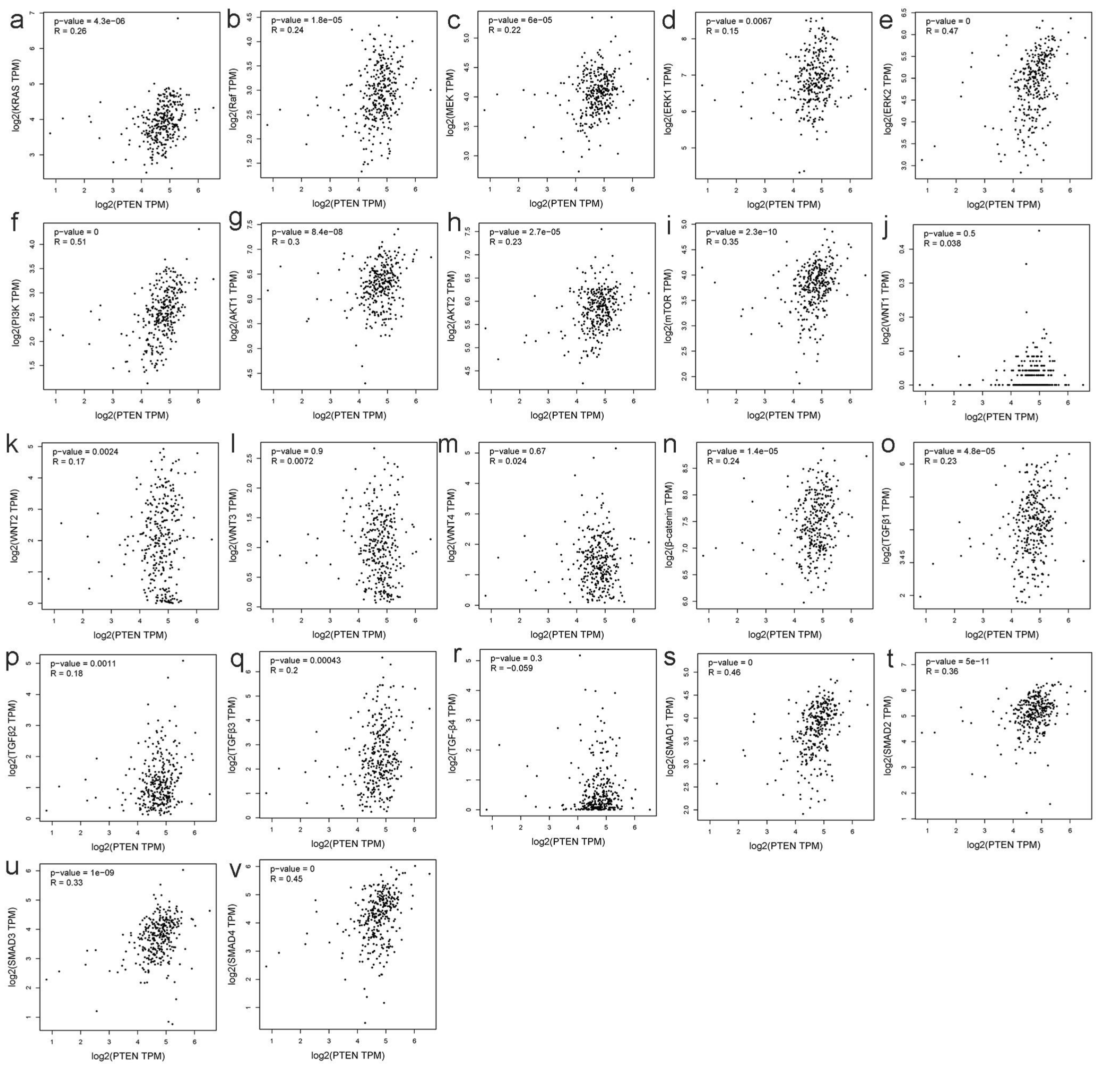
a
p-value = 4.3e-06
R = 0.26
log2(KRAS TPM)
log2(PTEN TPM)
b
p-value = 1.8e-05
R = 0.24
log2(Raf TPM)
log2(PTEN TPM)
c
p-value = 6e-05
R = 0.22
log2(MEK TPM)
log2(PTEN TPM)
d
p-value = 0.0067
R = 0.15
log2(ERK1 TPM)
log2(PTEN TPM)
e
p-value = 0
R = 0.47
log2(ERK2 TPM)
log2(PTEN TPM)
f
p-value = 0
R = 0.51
log2(PI3K TPM)
log2(PTEN TPM)
g
p-value = 8.4e-08
R = 0.3
log2(AKT1 TPM)
log2(PTEN TPM)
h
p-value = 2.7e-05
R = 0.23
log2(AKT2 TPM)
log2(PTEN TPM)
i
p-value = 2.3e-10
R = 0.35
log2(mTOR TPM)
log2(PTEN TPM)
j
p-value = 0.5
R = 0.038
log2(WNT1 TPM)
log2(PTEN TPM)
k
p-value = 0.0024
R = 0.17
log2(WNT2 TPM)
log2(PTEN TPM)
l
p-value = 0.9
R = 0.0072
log2(WNT3 TPM)
log2(PTEN TPM)
m
p-value = 0.67
R = 0.024
log2(WNT4 TPM)
log2(PTEN TPM)
n
p-value = 1.4e-05
R = 0.24
log2(β-catenin TPM)
log2(PTEN TPM)
o
p-value = 4.8e-05
R = 0.23
log2(TGFβ1 TPM)
log2(PTEN TPM)
p
p-value = 0.0011
R = 0.18
log2(TGFβ2 TPM)
log2(PTEN TPM)
q
p-value = 0.00043
R = 0.2
log2(TGFβ3 TPM)
log2(PTEN TPM)
r
p-value = 0.3
R = −0.059
log2(TGF-β4 TPM)
log2(PTEN TPM)
s
p-value = 0
R = 0.46
log2(SMAD1 TPM)
log2(PTEN TPM)
t
p-value = 5e-11
R = 0.36
log2(SMAD2 TPM)
log2(PTEN TPM)
u
p-value = 1e-09
R = 0.33
log2(SMAD3 TPM)
log2(PTEN TPM)
v
p-value = 0
R = 0.45
log2(SMAD4 TPM)
log2(PTEN TPM)

Figure S3

Table S3. The correction between PTEN and key molecules in signaling pathways of CRC.

Signaling pathways	Function	Key molecules	The correction with PTEN			Reference
			r^2	P	*-Log10(P-value)*	
KRAS signaling	Proliferation	KRAS [a]	0.26	4.3E-6	5.37	[35]
		Raf [a]	0.24	1.8E-05	4.74	
		MEK [a]	0.22	6E-05	4.22	
		ERK1 [b]	0.15	0.0067	2.17	
		ERK2 [c]	0.47	0	11.00	
PI3K signaling	Survival, suppressed apoptosis	PI3K [c]	0.51	0	11.00	[36]
		Akt1 [b]	0.3	8.4E-08	7.10	
		Akt2 [a]	0.23	2.7E-05	4.57	
		mTOR [b]	0.35	2.3E-10	9.64	
Wnt signaling	Proliferation, anti-apoptosis	Wnt1 [a]	0.038	0.5	0.30	[37]
		Wnt2 [b]	0.17	0.0024	2.62	
		Wnt3 [d]	0.0072	0.9	0.05	
		Wnt4 [d]	0.024	0.67	0.17	
		β-catenin [b]	0.24	1.4E-5	4.85	
TGF-β signaling	Proliferation	TGF-β1 [a]	0.23	4.8E-05	4.32	[36]
		TGF-β2 [b]	0.18	0.0011	2.96	
		TGF-β3 [c]	0.2	0.00043	3.37	
		TGF-β4 [d]	-0.059	0.3	0.52	
		SMAD1 [c]	0.46	0	11.00	
		SMAD2 [c]	0.36	5E-11	10.40	
		SMAD3 [c]	0.33	1E-09	9.00	
		SMAD4 [c]	0.45	0	11.00	

[a] $P < 0.05$; [b] $P < 0.005$; [c] $P < 0.0001$; [d] ns: no significance.

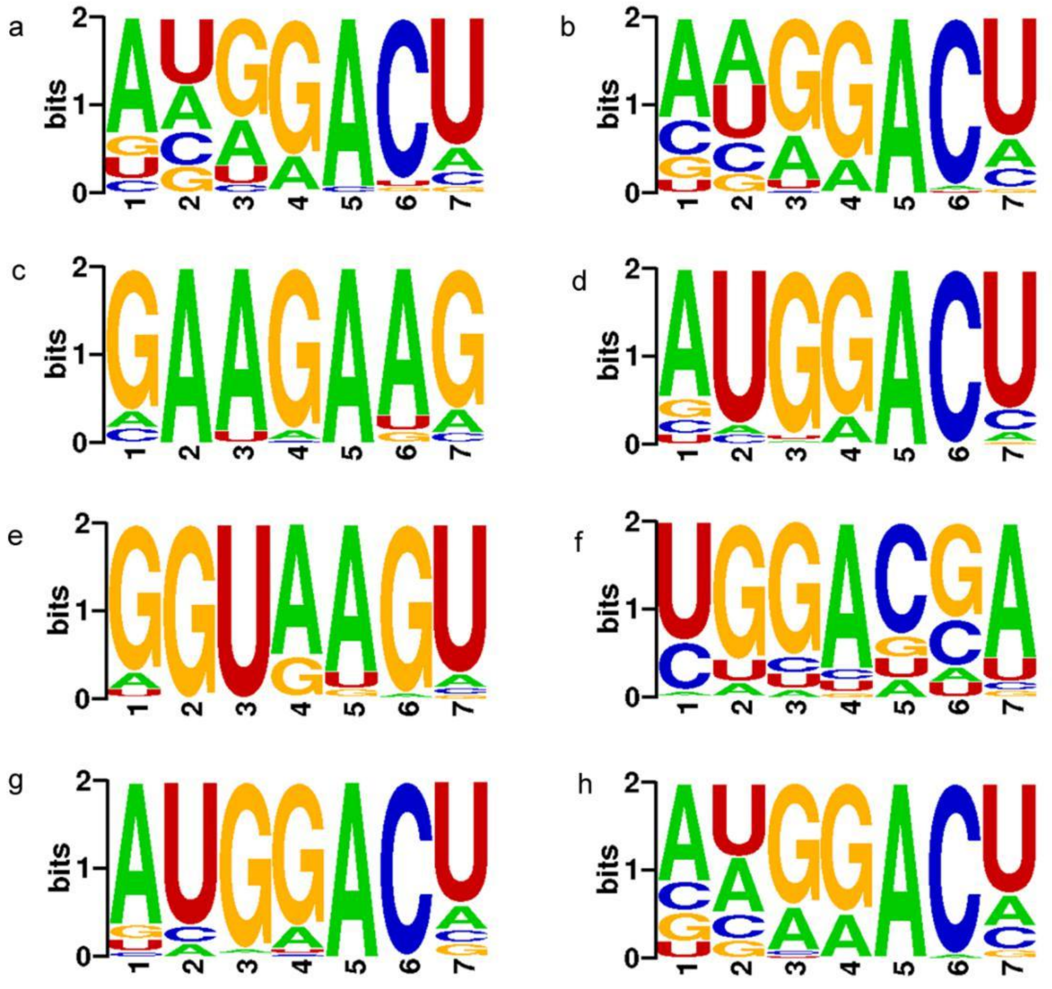

Figure S4

Table S4. All primers and siRNA used in cytological experiments.

Gene	Sequence
LINC01559	F: 5'-TGCAGACTCCTCAGGCTCATTC-3'; R: 5'-GTGGAAACTGATTTTCCACTTTACC-3'
miR-106b-3p	F: 5'-CGCGCGTAAAGTGCTGACAGT-3'; R: 5'-AGTGCAGGGTCCGAGGTATT-3'
PTEN	F: 5'-GTTTACCGGCAGCATCAAAT-3'; R: 5'-CCCCCACTTTAGTGCACAGT-3'
E-cadherin	F: 5'-CAGGTCTCCTCATGGCTTTGC-3''; R: 5'-CTTCCGAAAAGAAGGCTGTCC-3'
ZEB-2	F: 5'-TCCTCGTTTCTCCTTCTTTG-3'; R: 5'-GTTTGTTGGTCCTATCTCTTGT-3'
MMP-2	F: 5'-CCCTCAGAGCCACCCCTAAA-3'; R: 5'-CAGCCAGCAGTGAAAAGCCA-3'
MMP-3	F: 5'-TGGACAAAGGATACAACAGGG-3'; R: 5'-GCATCAAAGGACAAAGCAGG-3'
MMP-9	F: 5'-TTTGACAGCGACAAGAAGTGGG-3'; R: 5'-GTTCAGGGCGAGGACCATAGAG-3'
VEGFA	F: 5'-CCTCATCCTCTTCCTGCTCC-3'; R: 5'-CTTTCTCTTTTCTCTGCCTCCA-3'
hsa-miR-17-5p	F: 5’-CAAAGUGCUUACAGUGCAGGUAG-B-3’; R: 5’-ACCUGCACUGUAAGCACUUAGAG-3’
hsa-miR-20b-5p	F: 5'-TGCAGGTAGTTTTGGCATGA-3'; R: 5'-TCAACAAGAGATTTGTTATCCAAGAG-3'
hsa-miR-20a-5p	F: 5'-GCTGTCAACGATACGCTACGT-3'; R: 5'-GCCCGCTAAAGTGCTTATAGTG-3'
U6	F: 5'-CTCGCTTCGGCAGCACA-3'; R: 5'-AACGCTTCACGAATTTGCGT-3'
GAPDH	F: 5'-AACGGATTTGGTCGTATTGG-3'; R: 5'-TTGATTTTGGAGGGATCTCG-3'
hsa-miR-106b-5p-RT	GTCGTATCCAGTGCAGGGTCCGAGGTATTCGCACTGGATACGACATCTGC
si-LINC01559-3	5′-GCCATGACCTTGAATAAGT-3′
si-miR-106b-3p	5′-AUUUCACGACUGUCACGACUA-3′
si-METTL3	5′-GCACTTGGATCTACGGAAT-3′
si-NC	5′-CAGUACUUUGUGUAGUACAA-3′
linc01559 probe	5‘-DIG-GAACAACTGATCTGGGTCAGCCCTTCGT-DIG-3’
miR-106b-5p probe	5‘-DIG-ATCTGCACTGTCAGCACTTTA-DIG-3’

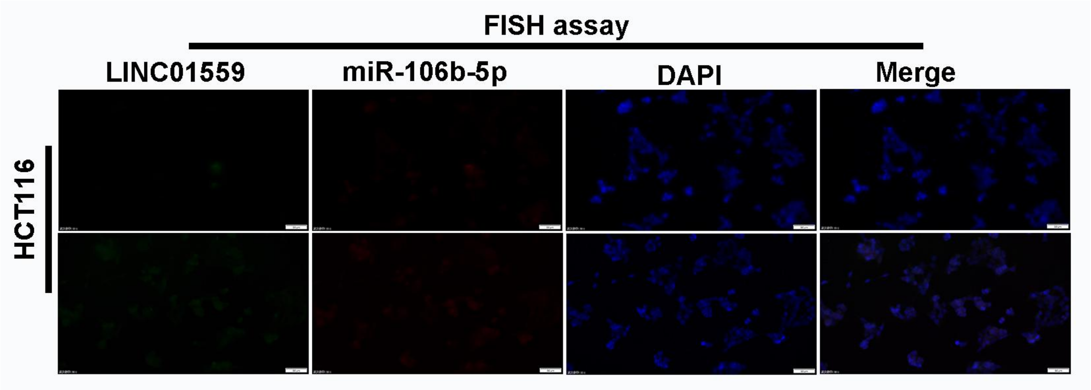

Figure S5

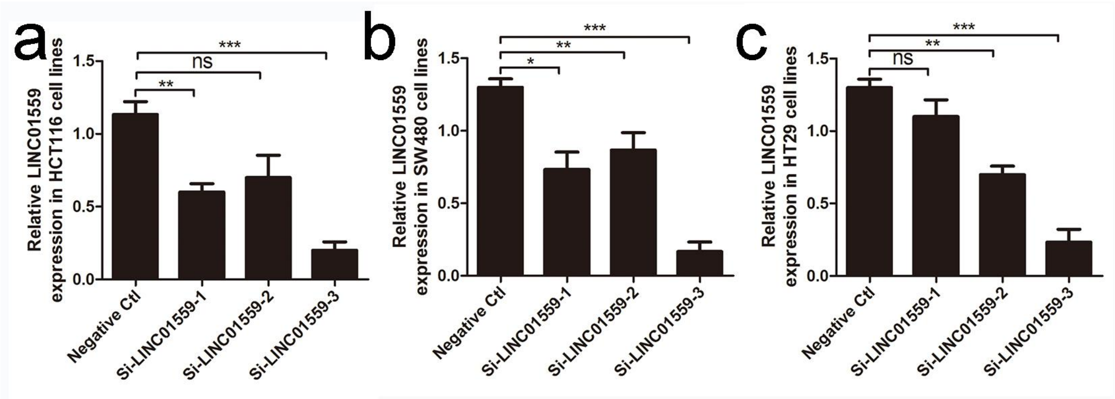

Figure S6

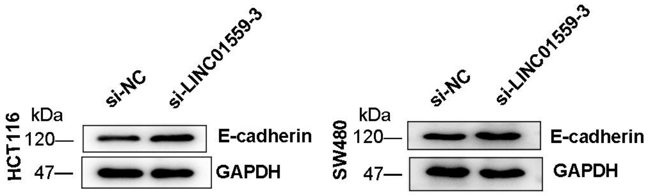

Figure S7

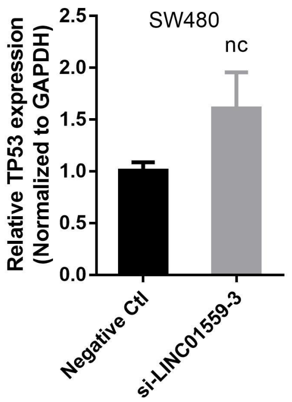

Figure S8

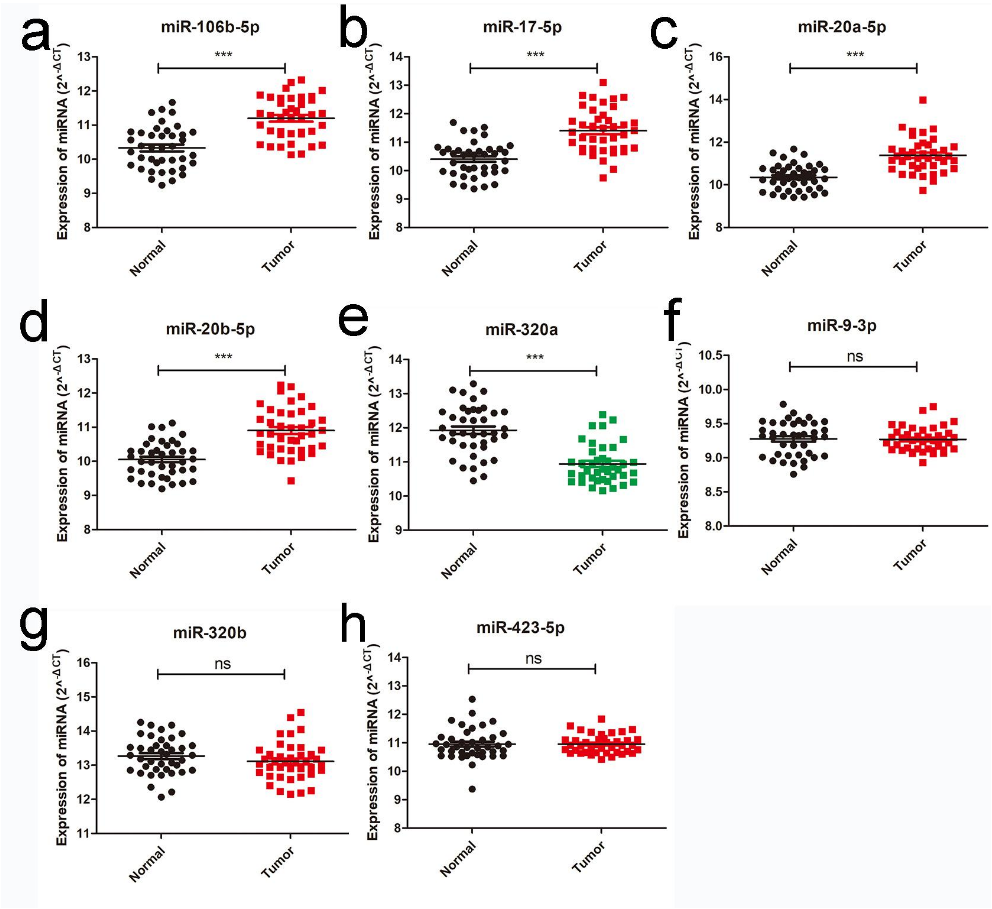

Figure S9

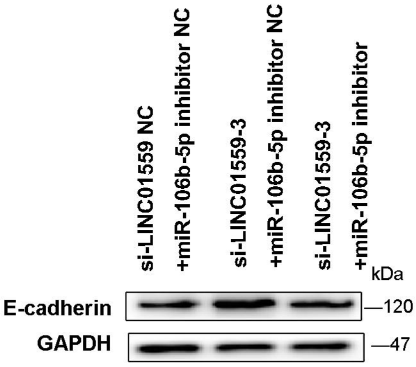

Figure S10

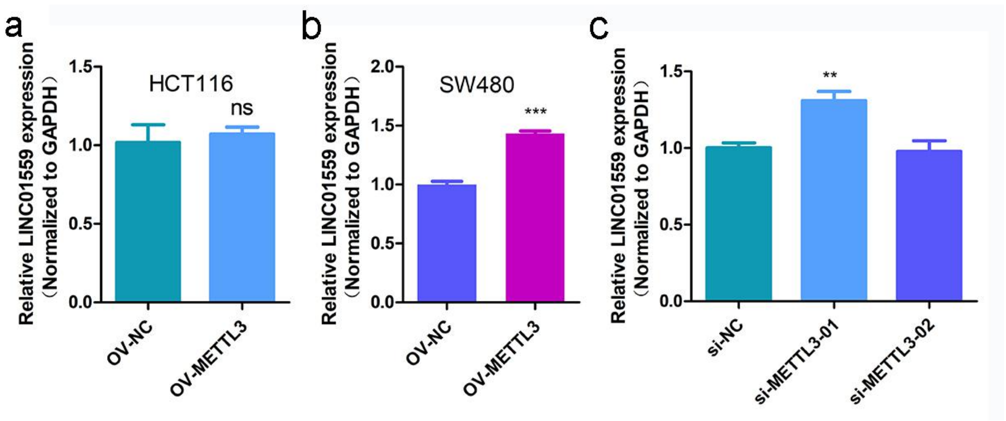
a
HCT116
ns
Relative LINC01559 expression (Normalized to GAPDH)
1.5
1.0
0.5
0.0
OV-NC
OV-METTL3
b
SW480
***
Relative LINC01559 expression (Normalized to GAPDH)
2.0
1.5
1.0
0.5
0.0
OV-NC
OV-METTL3
c
**
Relative LINC01559 expression (Normalized to GAPDH)
1.5
1.0
0.5
0.0
si-NC
si-METTL3-01
si-METTL3-02

Figure S11

Your job 'syCxeIOrkv' is finished

- Submission time: **Jan. 25, 2021, 9:50 p.m.**
- Prediction mode: **full, A549 cell**
- Analyzing RNA secondary structure: **NO**

Your FASTA sequence:

>NC_000007.14:c100094074-100093993 Homo sapiens chromosome 7, GRCh38.p13 Primary Assembly
CCUGCCGGGGCUAAAGUGCUGACAGUGCAGAUAGUGGUCCUCUCCGUGCUACCGCACUGUGGGUACUUGCUGCUCCAGCAGG

Please find your results below. You may also download the results in plain text from here. Note that the results will be automatically deleted after 72 hours!

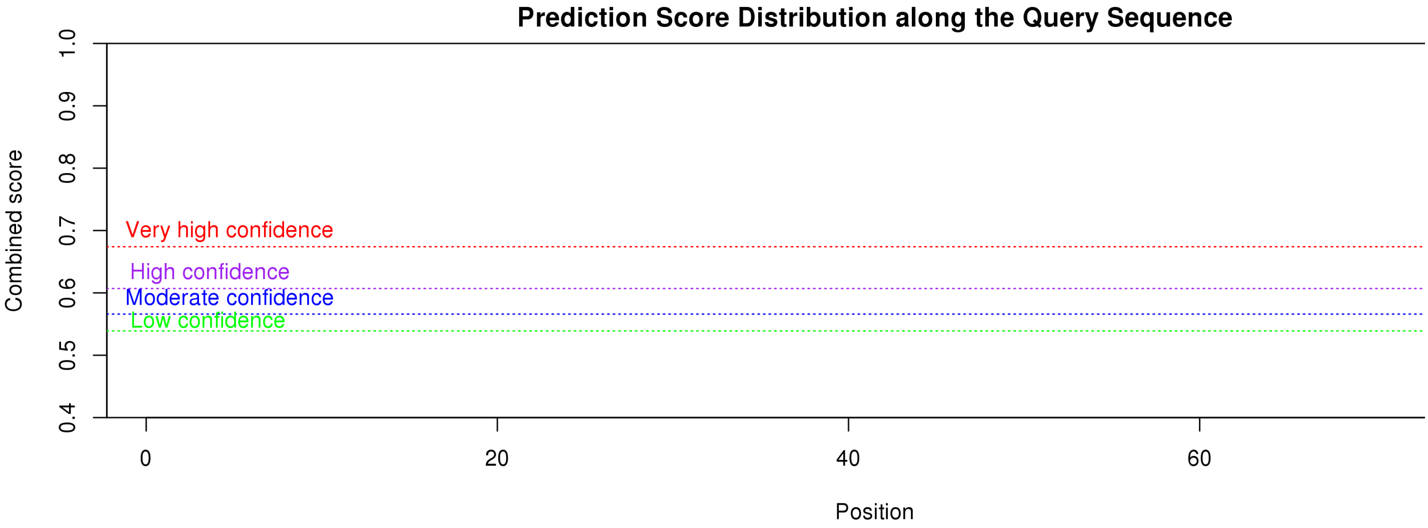

#	Position	Sequence context	Structural context	Local structure visualization	Score(binary)	Score(knn)	Score(spectrum)	Score(combined)	Decision
